# Algal polysaccharide Sacran-based conductive nanocomposites for ultrathin flexible and biodegradable organic electrochemical transistors

**DOI:** 10.1038/s41528-025-00436-1

**Published:** 2025-06-13

**Authors:** Katharina Matura, Christoph Putz, Sarka Hradilova, Katerina Polakova, Mihai Irimia-Vladu, Maiko Okajima, Tatsuo Kaneko, Martin Kaltenbrunner, Niyazi Serdar Sariciftci, Serpil Tekoglu

**Affiliations:** 1 Linz Institute for Solar Cells (LIOS) and Institute of Physical Chemistry, Johannes Kepler University Linz, Linz, Austria; 2 Division of Soft Matter Physics and Institute of Experimental Physics, Johannes Kepler University Linz, Linz, Austria; 3 Soft Materials Lab, Linz Institute of Technology, Johannes Kepler University Linz, Altenbergerstrasse 69, 4040, Linz, Austria; 4 Czech Advanced Technology and Research Institute (CATRIN), Regional Centre of Advanced Technologies and Materials (RCPTM), Palacký University Olomouc, Olomouc, Czech Republic; 5 School of Chemical and Material Engineering, Jiangnan University, Wuxi, China

**Keywords:** Biotechnology, Chemistry, Materials science, Nanoscience and technology

## Abstract

Organic electrochemical transistors (OECTs) have emerged as essential components in various applications, including bioelectronics, neuromorphics, sensing, and flexible electronics. Recently, efforts have been directed toward developing flexible and sustainable OECTs to enhance their integration into wearable and implantable biomedical devices. In this work, we introduce a novel PEDOT:Sacran bio-nanocomposite as a channel material for flexible and biodegradable OECTs. Sacran, a high-molecular-weight polysaccharide derived from blue-green algae, possesses exceptional ionic conductivity, water retention, and biocompatibility, making it a promising candidate for bioelectronic applications. We successfully fabricated ultrathin and flexible OECTs on poly(ethylene terephthalate) (PET) foils, achieving transconductance values up to 7.4 mS. The devices exhibited stable ion-to-electron transduction after mechanical deformation. The OECTs were further demonstrated on eco-friendly and biodegradable poly(lactic acid) (PLA) substrates, achieving a transconductance of 1.6 mS and undergoing enzymatic hydrolysis under controlled conditions. This study highlights the potential of Sacran-based conductive bio-nanocomposites in advancing sustainable bioelectronic devices.

## Introduction

Organic electrochemical transistors (OECTs) are advantageous for biological sensing, neuromorphics, and bioelectronics due to their high signal amplification at low operational voltage and facile integration in mechanically flexible circuits^1–9^. In recent years, there has been a growing interest in the development of flexible and biodegradable OECTs^10–14^. Flexible and stretchable OECTs are particularly appealing for their potential incorporation into soft, conformable devices. They can maintain high performance under mechanical deformation, a critical feature for applications in wearable electronics, soft robotics, and implantable biomedical devices^10,13,15^.

Among the channel materials utilized in OECTs, poly(3,4-ethylenedioxythiophene): polystyrene sulfonate (PEDOT:PSS) is the most widely used *p*-type polymer due to its high electrical conductivity, ease of processing, and good stability^16^. PEDOT:PSS has demonstrated promising performance in both vertical and flexible OECT devices^17,18^. PEDOT:PSS-based materials, when combined with elastic substrates and innovative processing techniques, have been successfully adapted to form stretchable devices^11,15,19–22^. By using additives, surfactants, and ionic liquids, these devices are capable of withstanding significant mechanical strain without a loss in performance^19,23^. Furthermore, the incorporation of biodegradable materials into the OECT structure holds promise for environmentally friendly applications, particularly in medical devices and bioelectronics, where devices need to degrade safely after use, eliminating the need for removal or long-term disposal^24–26^. In this context, a large set of biopolymers have been investigated to substitute the polystyrene sulfonate (PSS) component within the PEDOT:PSS composite to improve biocompatibility^27–30^. In particular, polysaccharide-based conductive biocomposites have exhibited high transconductance values in OECTs^29,30^.

The development of flexible and degradable OECTs remains a challenge due to the limited availability of conductive materials that offer both high electronic performance and environmental sustainability. To address this, we introduce Sacran, a high-molecular-weight polysaccharide extracted from blue-green algae, as a biocompatible and biodegradable polyanion to replace conventional synthetic polyelectrolytes in PEDOT-based composites. Sacran is from cyanobacterium *Aphanothece sacrum* (vernacular name, Suizenji Nori), which grows in mineral-rich river environments^31,32^. Suizenji Nori is a rare edible freshwater blue-green algae indigenous to Japan^33^. Previous studies have characterized the structure and properties of this unique bio-derived polysaccharide, highlighting its high content of anionic functional groups, such as carboxylate and sulfonate, which collectively constitute 32 mol% of the total sugar units^31,34^. Sacran is composed of several sugar units, including glucose (Glu), galactose (Gal), mannose (Man), xylose (Xyl), rhamnose (Rha), and fucose (Fuc), with a ratio of 25.9:11.0:10.0:16.2:10.2:6.9 as well as 20–25% of uronic acid^34,35^. This biopolymer possesses an exceptionally high molecular weight (*M*w = ~2 × 10⁷ g mol^−1^) and an extended chain length exceeding 8 µm, both of which, along with its high density of negative charges, contribute to its ionic absorption capabilities^31,32,35^. Despite its high molecular weight, it remains highly soluble in water due to its charged sugar residues. Sacran exhibits exceptional water retention, anti-inflammatory properties, and the ability to form hydrogels^32,36–38^. Flexible Sacran hydrogel films can be versatile, with high water content and elasticity^36^. These characteristics make it an attractive candidate for advanced medical applications, particularly in wound dressing, drug delivery systems, and cancer treatment^39^. Furthermore, Sacran exhibits inherent biocompatibility and has the potential to be used as scaffolds in tissue engineering and transdermal applications^36,37,40–42^.

Sacran’s intrinsic ionic conductivity, coupled with its biocompatibility, makes it an ideal matrix for embedding conductive fillers such as carbon nanomaterials or conducting polymers. In this context, its flexible chains can complex effectively with carbon nanotubes to form eco-friendly and functional bio-nanocomposites^34,43^. However, to date, there have been no reports of electrically conductive Sacran-based composites or their application in bioelectronics.

**Fig. 1:**
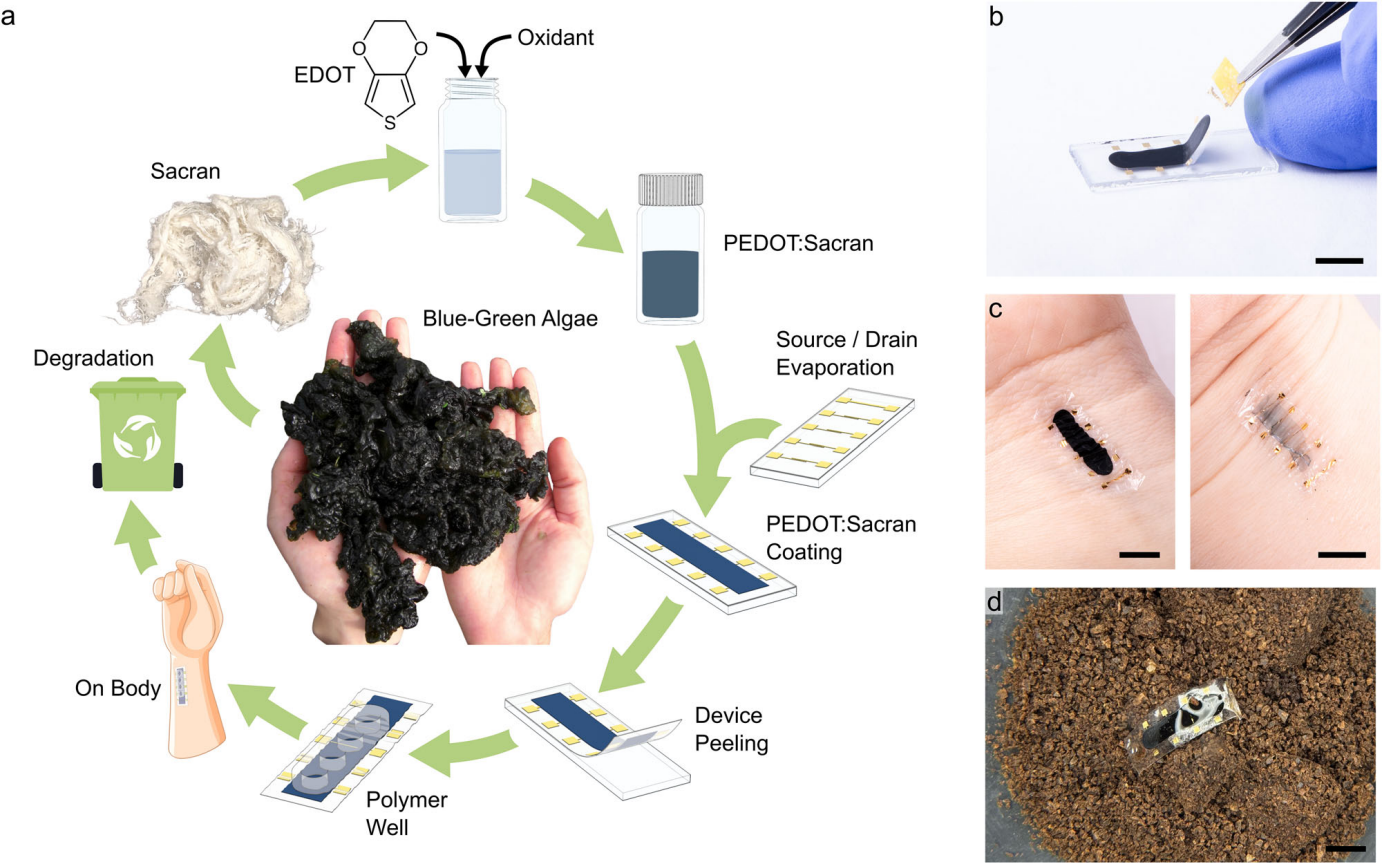
Schematic illustration and representative photographs of the ultrathin flexible, and biodegradable OECTs. **a** Schematic illustrations of the overall process: Extraction of Sacran from cyanobacteria *Aphanothece sacrum* (blue-green algae), synthesis of PEDOT:Sacran biocomposites, and device fabrication processes including source/drain (S/D) electrodes patterning, channel layer deposition, peeling off PET/PLA foil laminated on glass, applying polymer well to secure electrolyte solution during device operation, laminating OECTs on human skin, biodegradation of devices. (Resource image of Sacran, courtesy of Prof. Kaneko’s research group). **b** Device is peeling off from supporting platform to obtain a free-standing ultrathin OECTs. **c** Ultrathin flexible OECTs with a thick (left) and thin (right) polymer layer mounted on a compressed human wrist. **d** An exemplary image showing the device’s potential composting in soil. (Scale bars = 10 mm).

Herein, we present ultrathin, flexible, and biodegradable OECTs comprising a novel PEDOT:Sacran bio-nanocomposite as a channel polymer. The summary of the overall process is schematically illustrated as a circular flow diagram in Fig. 1. The strategy involves the extraction of the Sacran biopolymer from blue-green algae (Fig. 1a, middle) and the synthesis of Sacran-based conductive composites as the first step followed by the fabrication of ultrathin and flexible OECT devices on poly(ethylene terephthalate) (PET) foils. After the deposition of a drop-cast polymer film, the device is peeled off from the supporting platform to demonstrate the free-standing ultrathin OECTs (Fig. 1b). The final step of this process diagram refers to the degradation of transient devices built on poly(lactic acid) (PLA) substrates. Figure 1d visually references the potential compostability of PLA-based devices, supporting their broader environmental relevance.

The constructed devices on PET not only exhibit a mixed ionic-electronic conduction of the bio-nanocomposite with high transconductance values up to 7.4 mS but also preserve mechanical flexibility, with tensile strain surpassing 10% after pre-compression. As a proof of concept for skin-mountable devices, the ultrathin bendable devices were laminated on a human wrist to observe the conformability to the underlying surface. The device can fully follow the compression and stretching of skin (Fig. 1c). This demonstrates that the strategy imparts the resulting PEDOT:Sacran with high-fidelity ion-to-electron transduction, maintaining similar transconductance values even after bending and compression.

Ultimately, we successfully fabricated a series of OECTs with a novel channel polymer on different substrates without applying a photolithography technique. The study highlights the capability of producing OECTs on the eco-friendly and degradable material PLA, exhibiting a transconductance of up to 1.6 mS. The disposable OECTs device undergoes a microbial enzymatic hydrolysis under controlled conditions.

## Results

### Cyanobacterial polysaccharide ‘Sacran’-based bio-nanocomposites: synthesis and properties

The extracted ampholytic polysaccharide Sacran is mainly composed of various monosaccharides such as Glc, Gal, Man, Xyl, Rha, and Fuc with a composition of 25.9, 11.0, 10.0, 16.2, 10.2, and 6.9 as well as a 10 mol % sulfate and a 22 mol % carboxylic acid group to monosaccharide residues (detailed structure in Supplementary Fig. 1)^32^. These properties suggest that Sacran could be a promising candidate as an anionic polyelectrolyte, potentially facilitating strong electrostatic interactions with poly(3,4-ethylenedioxythiophene) (PEDOT), a positively charged conjugated polymer. Such interactions could promote charge stabilization and improve the processability of PEDOT, which is otherwise insoluble in water. This interaction potentially facilitates the formation of stable PEDOT microdispersions, further improving its suitability for water-based applications.

**Fig. 2:**
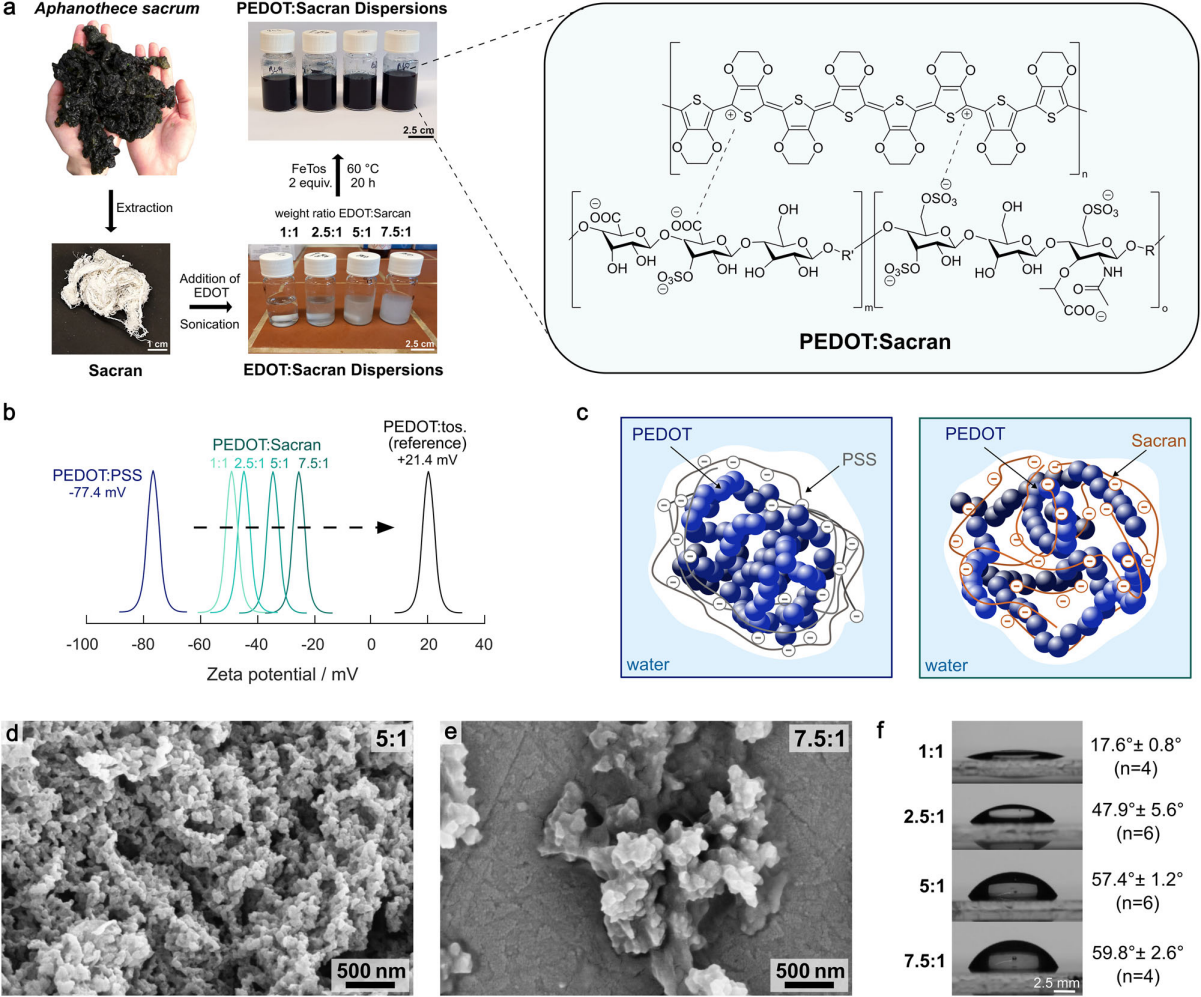
Cyanobacterial polysaccharide “Sacran”-based bio-nanocomposites: synthesis, structural and morphological properties. **a** Illustration of the overall synthesis procedure of the PEDOT:Sacran biocomposite, whereby EDOT monomer is added in different weight ratios to an aqueous Sacran solution and sonicated yielding a well-dispersed solution. During the oxidative chemical polymerization with EDOT and ferric tosylate (FeTos) the color of the dispersion changed from milky white to dark blue, whereby the color was retained for the solid powder form of the biocomposite. The shown molecular structures correspond to the final biocomposite material. **b** An illustration of the zeta potentials which are listed in Table 1. **c** Exemplary depiction of the biocomposite’s potential structure when incorporating different negatively charged counterions. **d**, **e** SEM images of PEDOT:Sacran with the weight-to-weight ratio of 5:1 and in 7.5:1, respectively (scale bars = 500 nm). **f** Contact angle measurement using 18 MΩ water on thin films of the PEDOT:Sacran composite in different weight ratios, whereby the data is given as average ± (SD) for *n* = *x* measurements.

After its isolation, the biopolymer was subsequently utilized for the preparation of the novel PEDOT:Sacran biocomposite, following an adapted version of the established methods for PEDOT:DNA and PEDOT:S-CNC composite^28,29^. For the oxidative chemical polymerization, EDOT was added into an aqueous solution of Sacran in different weight-to-weight ratios (1:1, 2.5:1, 5:1, 7.5:1) of EDOT:Sacran. The resulting conjugated PEDOT chains carry positive charges, which interact electrostatically with the negatively charged groups on Sacran through Coulomb forces. A respective graphical summary of the synthesis as well as the final product (in a dark blue aqueous dispersion or powder) are depicted in Fig. 2a with the detailed description in the “Methods” section.

The hydrodynamic diameter (*D*_*h*_), polydispersity index (PDI), and corresponding zeta potential (ζ-potential) of the various biocomposite samples are summarized in Table 1. The reference material, PEDOT:PSS (Clevios PH 1000), exhibited a *D*_*h*_ of 215 nm, whereas PEDOT:tosylate (PEDOT:Tos) had a significantly larger *D*_*h*_ of 680 nm. For the PEDOT:Sacran samples, the mean particle size increased with higher PEDOT content, ranging from 321 nm at a 1:1 weight ratio to 781 nm at a 7.5:1 ratio. This increase in particle size is likely due to aggregation effects, which become more pronounced as the PEDOT content rises. As we previously reported, pure Sacran can exhibit low *D*_*h*_ values in the range of ≈400 nm in aqueous solutions^44^. Ultrasonication plays a critical role in inducing conformational changes, leading to a reduction in the chain length of Sacran micro-fibrils^44^. After the chain scission of Sacran, the polymer can disperse well and remain stable in water.

**Table 1:** A summary of the measured zeta potentials and particle size distribution of the biocomposite with different initial EDOT:Sacran weight ratios

Material	EDOT:Sacran wt. ratio	ζ -potential (average ± SD)/mV	*D*_*h*_/nm	PDI	*n*
PEDOT:PSS (reference)	–	−77.4 ± 1.7	215 ± 94	0.427 ± 0.098	5
Sacran	–	−68.9 ± 4.0	–	–	–
PEDOT:Sacran	1:1	−50.6 ± 3.2	321 ± 29	0.373 ± 0.092	11
PEDOT:Sacran	2.5:1	−46.8 ± 3.5	370 ± 32	0.389 ± 0.119	19
PEDOT:Sacran	5:1	−36.5 ± 7.0	507 ± 103	0.395 ± 0.060	12
PEDOT:Sacran	7.5:1	−25.0 ± 1.5	781 ± 49	0.420 ± 0.076	6
PEDOT:Tos (control)	–	21.4 ± 0.5	680 ± 31	0.309 ± 0.068	5

A comparison of ζ-potentials revealed that pure Sacran carries a highly negative ζ-potential of −68.9 mV, whereas the PEDOT:Tos control sample exhibited a positive ζ-potential of +21.4 mV. The biocomposites followed a trend in which increasing PEDOT content resulted in a gradual shift in ζ-potential from −50.6 mV at the lowest ratio to −25.0 mV at the highest ratio. This trend is graphically represented in Fig. 2b. Fig. 2c provides a possible explanation for the correlation between ζ-potential and biocomposite structure. PEDOT:PSS is known to adopt a core-shell model, where a hydrophobic, conductive PEDOT core is surrounded by a hydrophilic, PSS-rich shell (Fig. 2c)^45,46^. This architecture enables PEDOT:PSS to form a stable aqueous dispersion^46^. In contrast, PEDOT:Tos lacks a stabilizing PSS shell and is instead doped with residual tosylate ions from synthesis, leading to poor dispersion, aggregation, and precipitation of the material. The PEDOT:Sacran biocomposites likely adopt a more intermixed structure, where the polysaccharide and conductive PEDOT phases integrate more effectively, resulting in moderate dispersibility across all ratios.

Scanning electron microscopy (SEM) was used to analyze the surface morphology of the prepared nanocomposites (Fig. 2d, e). The PEDOT:Sacran composite with a lower wt. ratio (5:1) shows a more interconnected, globular morphology with smaller, uniformly distributed particles. In contrast, the 7.5:1 sample exhibits a coarser, more granular surface, likely resulting from larger aggregates and isolated clusters. This morphological difference is attributed to the increased concentration of PEDOT in the composite^47^. The surface wetting properties of the composite thin films are further analyzed using contact angle measurements. For all measurements, the biocomposite dispersion as well as the reference (PEDOT:PSS) were prepared with additives, whereby 94.5% (v/v) PEDOT:Sacran dispersion was mixed with 0.5% (v/v) DBSA, 5% (v/v) Glycerol and finally 1% (v/v) GOPS with respect to the total volume of the dispersion^48^. A clear increase in contact angle was observed, rising from 17.6 ± 0.8° at a 1:1 ratio to approximately 59.8 ± 2.6° at a 7.5:1 ratio, indicating an enhancement in hydrophobicity of the thin film. The contact angle of PEDOT:PSS (Supplementary Fig. 2) with 46.3 ± 7.2° is in accordance with literature values^49^.

### Spectroscopic and thermal characterization of PEDOT:Sacran bio-nanocomposites

**Fig. 3:**
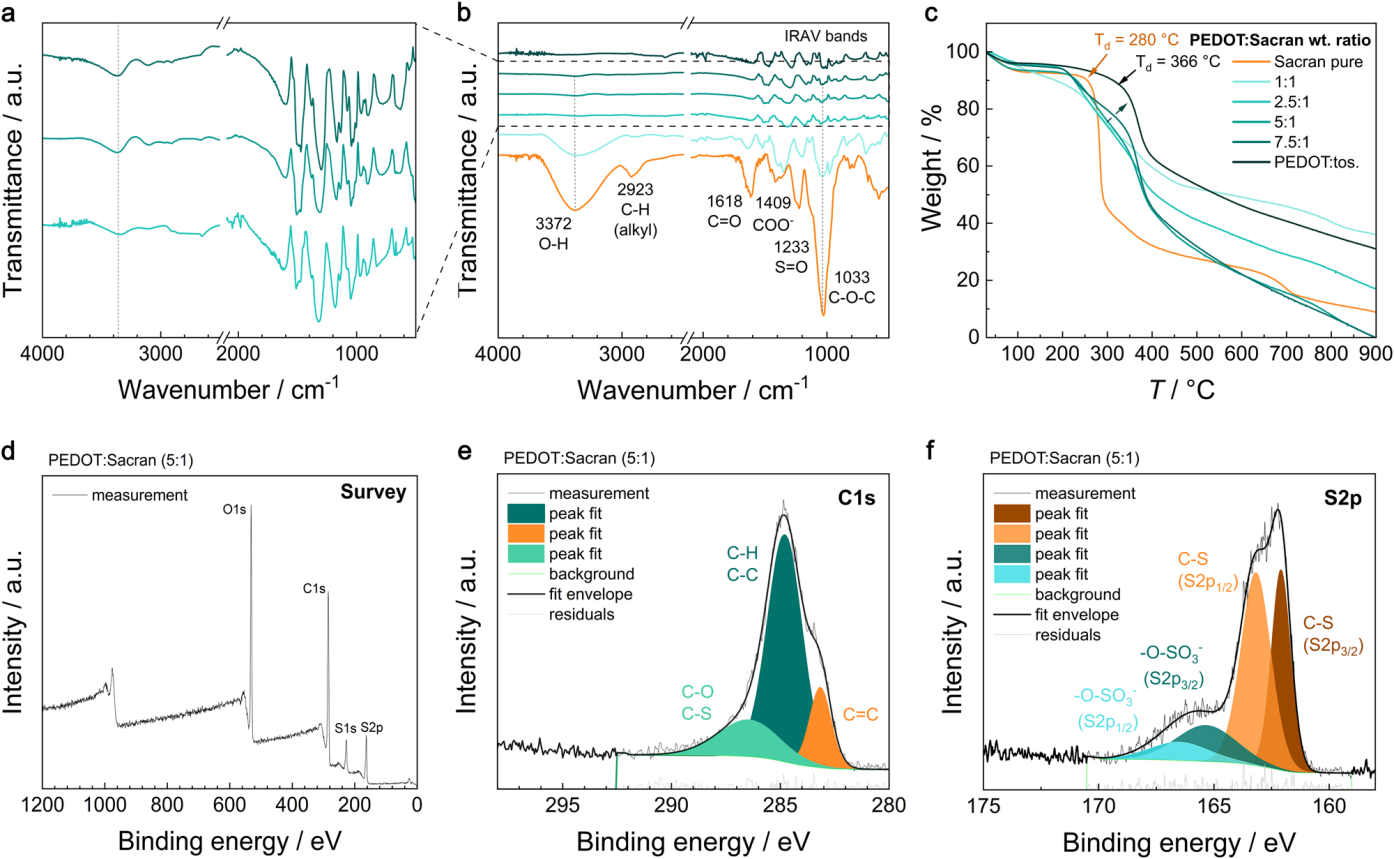
Spectroscopic and thermal characterization of PEDOT:Sacran bio-nanocomposites. **a** Magnified view of the ATR-FTIR spectra for the PEDOT:Sacran biocomposites at weight ratios of 2.5:1, 5:1, and 7.5:1 (EDOT:Sacran), highlighting the O–H stretching vibrations and IR-active vibrational (IRAV) bands. **b** Full ATR-FTIR spectra of the reference materials—Sacran (orange line) and PEDOT:Tos (black line)—alongside the biocomposites, with increasing PEDOT concentration indicated by a color gradient from light to dark petrol. **c** TGA diagrams for the reference materials as well as the biocomposites in different weight ratios. **d** XPS survey spectrum of PEDOT:Sacran (EDOT:Sacran 5:1) with the respective high-resolution scan for **e** C1s and **f** S2p XPS signals.

To examine the material characteristics and to enlighten the molecular structure of the composite materials measurements were conducted using Fourier-transform infrared spectroscopy (FTIR) in ATR (attenuated total reflection) mode, X-ray photoelectron spectroscopy (XPS), and thermal gravimetric analysis (TGA) (Fig. 3).

The results of the ATR-FTIR spectroscopy are shown for the reference materials as well as the PEDOT:Sacran biocomposite prepared from different initial weight ratios of EDOT:Sacran (Fig. 3a, b). From the FTIR analysis changes in the chemical composition, as well as structural motives, of the biocomposite with respect to the reference materials could be confirmed. Under the observation of the FTIR spectrum of pure Sacran the broad peak at 3372 cm^−1^ can be clearly attributed to the presence of O-H stretching vibrations within hydroxyl groups of the sugar chains^29,50,51^. The bands at 2923 cm^−1^ are characteristic for C-H stretching for alkyl groups within the Sacran backbone^50^. Furthermore, the peaks at 1618, 1409 and 1233 cm^−1^ and can be linked to Sacran-characteristic transmission bands of C=O, COO^−^ and S=O, respectively^50^. The peak at 1033 cm^−1^ can be assigned to C-O-C vibrations in both Sacran owing to the glycosidic ether bridges as well as in the PEDOT backbone^50,52^. When observing the FTIR spectrum of PEDOT, the infrared-active vibrational (IRAV) bands, corresponding to the doped state of PEDOT, can be clearly observed within the range of 1600–500 cm^−1^ ^53,54^. The biocomposite shows characteristic bands of both reference materials, proving the successful preparation of the PEDOT:Sacran biocomposite.

XPS analysis reveals the elemental compositions of the biocomposite (Fig. 3d, f). The XPS spectra confirmed the presence of the C, O, and S elements in the biocomposite material within all investigated initial weight ratios of PEDOT:Sacran. By analyzing the peak deconvolution of the C1s spectrum, the peak at 283.3 eV, 284.8 eV and 286.3 eV can be respectively assigned to the C=C–O bond of the EDOT unit in the β-position, the C–C bond of the aromatic ring structure of PEDOT as well as sugar units of Sacran and the C–O–C bond in the ethylene bridge of PEDOT^55^. The analysis of the S2p spectrum shows the S2p_3/2_-S2p_1/2_ spin-split doublet corresponding to PEDOT, with values of 162.1 eV and 163.2 eV, respectively^56,57^. In contrast, the peaks at 165.0 eV and 166.6 eV could be attributed to the S2p_3/2_-S2p_1/2_ spin-split doublet of the –O–SO_3_^−^ groups of uronic acid groups within the Sacran chain. A complete elemental analysis with the calculation of C, O, and S ratios can be found in Supplementary Table 2. No residual Fe²⁺ ions from the oxidant were detected after the polymerization of the initial EDOT for EDOT:Sacran ratios of 2.5:1, 5:1, and 7.5:1 (Supplementary Fig. 4). However, the lowest EDOT:Sacran ratio (1:1) exhibited residual Fe²⁺ ions, likely due to Sacran’s ability to absorb metal ions^31,34^.

The thermal properties of the PEDOT:Sacran biocomposites were analyzed using thermogravimetric analysis (TGA) under a nitrogen atmosphere, monitoring weight loss as a function of temperature, as depicted in Fig. 3c. This approach also allows us to confirm the presence of Sacran within the biocomposites by comparing their degradation temperatures with the reference materials. The TGA curves reveal that biocomposites with EDOT:Sacran weight ratios of 2.5:1, 5:1, and 7.5:1 remain thermally stable up to 200 °C. The initial weight loss observed up to 100 °C corresponds to the evaporation of water within the material. Pure Sacran begins to degrade at approximately 276 °C, while PEDOT:Tos exhibits thermal degradation starting around 339 °C, with both values being in agreement with the literature values^47,58–60^. In all biocomposites, two distinct degradation onset temperatures are present, aligning with those of the reference materials, thereby confirming the incorporation of Sacran within the biocomposites.

### Fabrication and characterization of PEDOT:Sacran-based OECTs on glass substrates

The applicability of the prepared biocomposite as an organic mixed ionic-electronic conductor (OMIEC) was tested within OECTs. First, the electrical conductivity of thin-films was investigated (results are shown in Table 2). Here, a trend is observed in which increasing the PEDOT content leads to a corresponding increase in conductivity, reaching 1.88 S cm^−1^ for the highest PEDOT:Sacran ratio (7.5:1). This value was obtained for a drop-cast film (approximately 4–8 µm thick) prepared from a dispersion containing 94.5% (v/v) PEDOT:Sacran, 0.5% (v/v) DBSA, 5% (v/v) glycerol, and finally 1% (v/v) GOPS with respect to the total dispersion volume. These additives are commonly used in OECTs fabrication to stabilize films and prevent delamination^48^. The reported electrical conductivity is comparable to previously studied biocomposites incorporating polysaccharides as negatively charged counterions, such as PEDOT:sulfated-cellulose nanocrystals with 5 S cm^−1^ ^29^ or PEDOT:pectin with <0.01 S cm^−1^ ^27,30^.

**Table 2:** The overview of determined device parameters of OECTs and the measured conductivity of corresponding polymer thin-films

Material	EDOT: Sacran wt. ratio	Film conductivity^a^/S cm^−1^	g_max_/mS	*g*_max_(NR)/S cm^−1^	*V*_g,max_/V	On/Off ratio	*N*^b^
PEDOT:Sacran	1:1	2.4 × 10^−3^ ± 1.5 × 10^−4^	0.0026	1.4 × 10^−3^	−0.11	5.3	1
PEDOT:Sacran	2.5:1	0.244 ± 0.085	0.03 ± 0.01	0.02 ± 0.01	−0.22 ± 0.07	15 ± 7	10
PEDOT:Sacran	5:1	1.31 ± 0.14	0.18 ± 0.05	0.11 ± 0.02	−0.31 ± 0.06	38 ± 13	8
PEDOT:Sacran	7.5:1	1.88 ± 0.21	0.08 ± 0.07	0.05 ± 0.03	−0.34 ± 0.08	32 ± 15	10
PEDOT:PSS (PH 1000)	–	357 ± 1.7	33.1 ± 5.3	22.5 ± 5.2	0.21 ± 0.07	8 ± 4	12

^a^Electrical conductivity was measured per biocomposite ratio from two substrates with three measurement points on each substrate (*n* = 6).

^b^Number of devices included for the evaluation of the steady-state transfer characteristics.

**Fig. 4:**
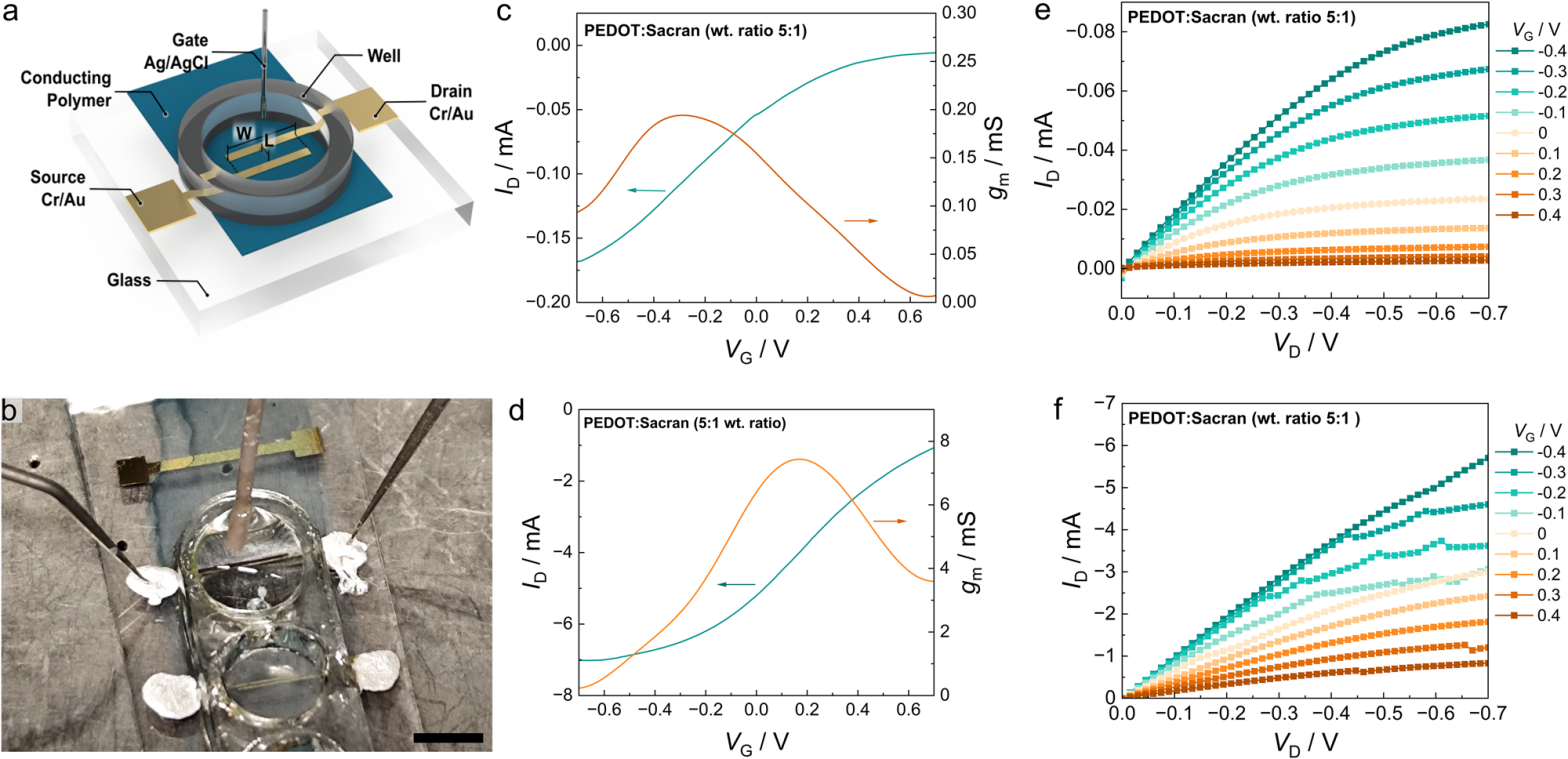
Fabrication and characterization of PEDOT:Sacran-based OECTs on glass substrates. **a** A schematic depiction of the device structure of an PEDOT:Sacran based OECT on glass substrate, including Cr/Au source and drain electrodes, PEDOT:Sacran as channel material, the polymer well for the electrolyte and the non-polarizable Ag/AgCl gate electrode. *W* and *L* are referred to as width and length. **b** A photo of an OECT under operation (Scale bar = 2 mm). **c** Transfer characteristics and the corresponding transconductance curve (for *V*_D_ = −0.7 V) and **e** the corresponding output characteristics for OECT devices based on a 5:1 wt. ratio PEDOT:Sacran dispersion (*W* = 2.1 mm, *L* = 65 µm, *d* = 480 nm). Steady-state electrical characterization of drop-cast film PEDOT:Sacran-based OECTs: **d** Transfer characteristics and the corresponding transconductance curve (for *V*_D_ = −0.7 V) and **f** the corresponding output characteristics for an OECT device based on a 5:1 wt. ratio PEDOT:Sacran dispersion (*W* = 2.2 mm, *L* = 47 µm, *d* ~ 7.5 μm). Smoothing is applied for all transconductance curves: 10 pts FFT.

To evaluate the ion-to-electron transduction capability of the synthesized biocomposites for bioelectronic applications, OECTs were fabricated on glass substrates using the device configuration shown in Fig. 4a. The steady-state transfer characteristics (*I*_D_ vs. *V*_G_) for the best performing wt. ratio of 5:1 for PEDOT:Sacran are presented in Fig. 4c, d with the channel thicknesses of 480 nm and ~7.5 µm, respectively. The corresponding transconductance (${g}_{\text{m}}=\partial {I}_{\text{D}}/\partial {V}_{\text{G}})$ is shown alongside the transfer curve. A statistical analysis of the maximum transconductances (*g*_max_) values for OECTs with different wt. ratios is summarized in Table 2. To account for variations in channel geometry and layer thickness, the normalized maximum transconductance *g*_max_ (*g*_max_(NR)) is also provided, calculated using the following formula:

1 $${g}_{max}({\rm{N}}{\rm{R}})={g}_{{\rm{m}}{\rm{a}}{\rm{x}}}\cdot \frac{L}{d\cdot W}$$

where *L* is the channel length, *d* is the film thickness, and *W* is the channel width^61^. This normalization aims to eliminate any concentration-dependent thickness variations arising from different PEDOT:Sacran ratios.

In general, for all devices, it is clearly evident that the absolute value of drain current (*I*_D_) decreases as the gate voltage (*V*_G_) becomes more positive. This occurs because positively charged ions migrate from the electrolyte solution into the PEDOT layer, reducing its conductivity by de-doping the material. As a result, the PEDOT layer transitions to a low-conductivity state, approaching zero conductivity^62^. The devices function in depletion mode, exhibiting the characteristics of *p*-type OMIEC channel materials. For all PEDOT:Sacran ratios (1:1, 2.5:1, 5:1, and 7.5:1), the OECT devices could be switched “off” by increasing the gate voltage to 0.6 V. An exemplary depiction of the steady-state transfer characteristics for the ratios 1:1, 2.5:1, and 7.5:1 ratios is provided in Supplementary Fig. 5.

PEDOT:PSS, the most commonly used reference channel material in the field of bioelectronics, was also included in this study for comparison. Interestingly, the highest *g*_max_ and *g*_max_(NR) values were obtained for the 5:1 ratio of PEDOT:Sacran, reaching 0.18 mS and 0.11 S cm^−1^, respectively. In comparison, the PEDOT:PSS OECTs showed significantly higher values of 33.1 mS and 22.5 S cm^−1^ (Supplementary Fig. 6). The output characteristics (*I*_D_ vs. *V*_D_) of the devices with the wt. ratio 5:1 is depicted in Fig. 4e and for the other wt. ratios in Supplementary Fig. 5, measured at constant *V*_G_. It is evident that only devices with PEDOT:Sacran with the wt. ratios of 5:1 and 7.5:1 reach near saturation at applied positive gate voltages. To further enhance transconductance, drop-cast PEDOT:Sacran-based OECTs were also characterized, as shown in Fig. 4f. The *g*_max_ strongly depends on the channel geometry and is directly proportional to *WdL*^−1^ (*g*_max_ ∝ *WdL*^−1^)^61^. Here, the transconductance of 7.4 mS was achieved by increasing the channel thickness to 7.5 μm. The gate current characteristics of the optimized wt. ratio of 5:1 for PEDOT:Sacran can be found in Supplementary Fig. 7a, b.

### Device performance of ultrathin and flexible PEDOT:Sacran-based OECTs on PET substrates

**Fig. 5:**
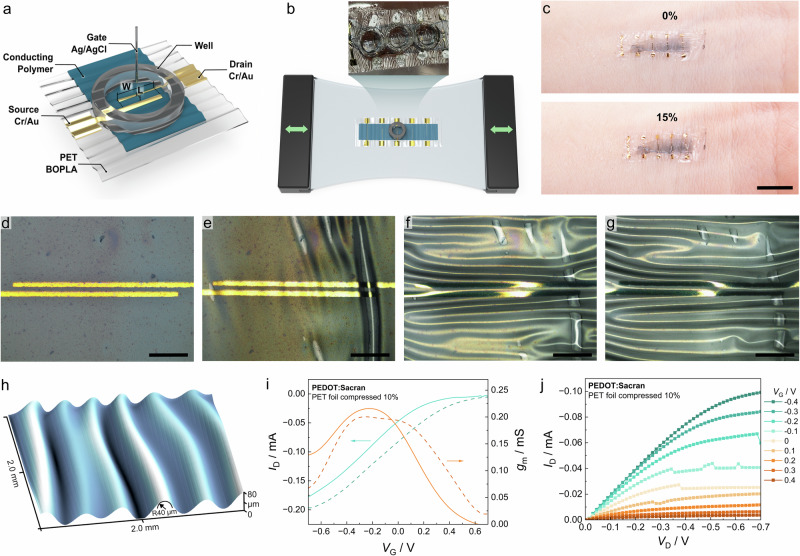
Device performance of ultrathin and flexible PEDOT:Sacran-based OECTs on PET substrates. **a** The sketch represents the device structure of an PEDOT:Sacran based OECT on a 1.4 µm PET foil, including Cr/Au source and drain electrodes. **b** Illustration of the compression test unit, whereby the OECT device was laminated on a pre-stretched acrylic elastomer tape to compress the substrates simultaneously while measuring the steady-state characteristics. The inset shows a substrate during 10% compression. **c** Ultrathin flexible OECT mounted on human skin with initial and 15% compression (Scale bar = 10 mm). A non-toxic solvent free adhesive was used to laminate the device on the skin. Optical microscope images (×5 magnification) of an exemplary device (Scale bars = 500 µm): **d** with the PET foil still adhered on glass, **e** the PET foil after placing it on a fully expanded elastomer tape, **f** the PET substrate under 5% compression and **g** the PET substrate under 10% compression. **h** Surface profile of a PET foil under 10% compression. Steady-state electrical characterization of PEDOT:Sacran-based thin-film OECT on PET foil under 10% compression. **i** Transfer characteristics and the corresponding transconductance curve (for *V*_D_ = −0.7 V) for a device on the 10% compressed PET foil (lines) in comparison to device on glass (dashed line, data copied from Fig. 5b), and **j** the corresponding output characteristics for the 10% compressed PET foil substrate OECT device based on a 5:1 wt. ratio PEDOT:Sacran dispersion (*W* = 2.2 mm, *L* = 31 µm, *d* ~ 600 nm). Smoothing method is applied for transconductance curves: 10 pts Savitzky Golay.

The adaptation of flexible/stretchable substrates is a widely used strategy to achieve mechanically compliant OECTs. Poly(ethylene terephthalate) (PET) and poly(lactic acid) (PLA) are commonly employed as flexible substrates; however, these polymers do not exhibit significant stretchability due to their thickness (~20 µm)^10^. To investigate the applicability of the novel biocomposite with an optimized weight ratio in flexible OECTs, we fabricated devices using ultrathin 1.4 µm PET substrates (Fig. 5a). After depositing thin-film or drop-cast polymer films, the devices were peeled off from the supporting glass substrate and transferred onto a pre-stretched acrylic elastomer tape to evaluate their performance under mechanical strain (Fig. 5b). Tensile testing of ultrathin PET and PLA substrates revealed clear limitations in their stretchability (Supplementary Fig. 8a). While both materials are flexible, their mechanical responses differ significantly. The PET samples demonstrated approximately twice the strain at failure (~20%) compared to PLA, which shows a more brittle behavior with lower elongation. Figure 5c shows the ultrathin 1.4 µm OECT device with excellent conformability to the skin. The ultrathin flexible OECT was mounted on human skin under various compression ratios of 0, 15, 20, and 30% (in Supplementary Fig. 9). Such devices, with a thickness of less than 5 µm have the advantage of high compliance on the skin, which is favorable for wearable technologies^19^.

An ultrathin PET sample, subjected to approximately 10% compressive strain, was analyzed using a stylus profilometer. The three-dimensional map of the wrinkle morphology reveals the formation of sinusoidal folds with wavelengths ranging from 300 µm to 500 µm and peak amplitudes of up to 100 µm (Fig. 5h). This significant mechanical deformation causes the OECT channel to undergo multiple bends, with radii of curvature as small as 40 µm (Fig. 5h) or potentially even smaller.

To evaluate the mechanical stability, we tested OECTs under mechanical deformations. The maximum transconductance of 0.2 mS was almost identical compared to OECTs on glass (with thin-film polymer channel). A small difference (~0.02 mS) in the transconductance values can be related to batch-to-batch variations in the channel geometry and film thicknesses. The subsequent wrinkles caused negligible changes in the transconductance values at pre-stretched (0%) and compressed (5%, 10%) state of the substrates. The *g*_max_ of 0.2 mS is also achieved for PEDOT:Sacran ultrathin flexible OECTs (Fig. 5i) in a compressed state after stretching. A summary of device performance of OECT at the pre-stretched and compressed state (5% and 10%) is shown in Table 3. Assuming a channel layer thickness of approximately 600 nm (on PET substrate), the normalized transconductance is calculated to be 0.08 S cm^−1^, based on the values in Table 3. This performance is comparable to the 0.83 S cm^−1^ reported by Liao et al., who employed a 50 µm flexible PET substrate with PEDOT:PSS and a PANI/Nafion–graphene/Pt gate electrode^63^. Takemoto et al. reported a maximum transconductance of approximately 0.1 mS during bending tests on ultrathin (2 µm) PEDOT:PSS-based OECTs^19^. In comparison, our study achieved a *g*_max_ of 0.2 mS during compression tests on ultrathin (1.4 µm) PEDOT:Sacran-based OECTs. Similarly, Park et al. achieved a transconductance of 0.8 mS in OECTs fabricated on parylene substrates with PEDOT:PSS as the channel material under 33% compressive strain^64^. A selection of studies utilizing flexible and/or stretchable substrates, such as PET and PLA, for OECT applications is summarized in Supplementary Table 3.

**Table 3:** Summary of the OECTs measured in the initial (pre-stretched) state and during compression of the substrate (5% and 10%)

Step	Percentage compression (substrate)/%	*g*_max_/mS	*V*_g,max_/V	*n*
Pre-stretched	0	0.20 ± 0.09	−0.36 ± 0.05	6
Compressed	5	0.20 ± 0.10	−0.39 ± 0.04	4
Compressed	10	0.20 ± 0.03	−0.33 ± 0.11	5

In the first deformation in the bending state (diameter = ~1 cm), the maximum transconductance was obtained 2.3 mS (Supplementary Fig. 6a) for OECTs with thick channel polymer (~5 µm). Mechanical instability during deformation, such as cracking in the polymer channel, can be minimized by modifying the device structure—specifically by reducing the channel area and using solid-polymer electrolytes to limit strain effects^65^.

### Transient and biocompatible PEDOT:Sacran-based OECTs on PLA substrates

**Fig. 6:**
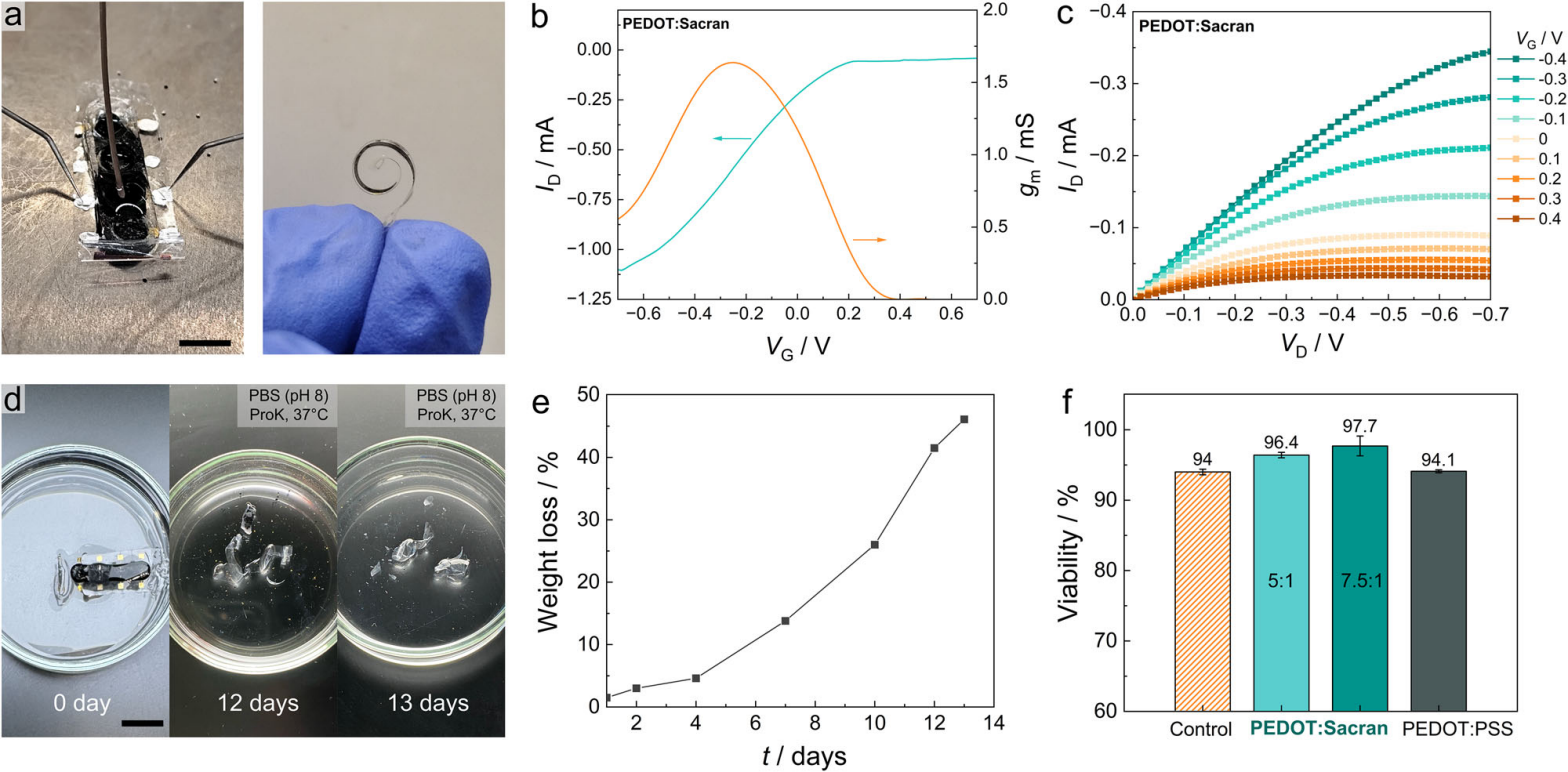
Transient and biocompatible PEDOT:Sacran-based OECTs on PLA substrates. **a** Picture of an PEDOT:Sacran (drop-cast film) based OECT fabricated on a 20 µm PLA foil during measurement. Side view of the flexible PLA substrate without the polymer well. **b** Transfer characteristics and the corresponding transconductance curve (for *V*_D_ = −0.7 V) and I), and **c** the corresponding output characteristics for the OECT device based on a 5:1 wt. ratio PEDOT:Sacran dispersion on the PLA foil (*W* = 2.2 mm, *L* = 35 µm, *d* ~ 9.5 μm). **d** Degradation of transient device under controlled conditions by following immersion in 0.05 M PBS (pH 8) solution incl. 2 mg proteinase K at an elevated temperature (37 °C) for accelerated degradation. **e** Weight loss (%) for enzymatic biodegradation at different time intervals. **f** Evaluation of biocompatibility of PEDOT:Sacran samples according to ISO standard. Note: Cell medium applied on the studied samples for 24 h was discarded, reused and cultivated with L929 cells seeded in the commercial plastic chambers for another 24 h.

By focusing on the high potential of the biocomposite for the development of sustainable and eco-friendly bioelectronics, we fabricated intrinsically degradable OECTs (Fig. 6a) with a thick channel layer ~9.5 μm on PLA substrates using the same device stack in Fig. 5a. Figure 6b, c presents the steady-state transfer characteristics of the OECT device using the optimized PEDOT:Sacran weight ratio of 5:1. The maximum transconductance of 1.6 mS was obtained and graphed alongside the corresponding transfer curve. The output characteristics recorded at constant *V*_G_, showed that PEDOT:Sacran (5:1) are reaching saturation at lower *V*_D_ and for nearly all measured *V*_G_. The gate current characteristics of PEDOT:Sacran-based OECTs on flexible and biodegradable substrates are included in Supplementary Fig. 7c–e. For fully biodegradable devices, the polymer wells can be readily replaced with biodegradable materials such as starch-based bioplastics. Therefore, the biodegradable gelatine wells were combined with the device architecture to create fully degradable OECTs as a proof-of-concept study. The maximum transconductance was calculated in the range of 0.2–0.3 mS (Supplementary Fig. 10). Fumeaux et al. reported PEDOT:PSS-based OECTs fabricated entirely from transient materials on PLA, achieving a transconductance of approximately 0.3 mS^24^, which is comparable to the values obtained in our study. Additionally, Supplementary Table 3 provides an overview of other OECT devices fabricated on biodegradable substrates such as PLA and poly(lactic-co-glycolic acid) (PLGA). Notably, various studies have investigated biodegradable, electrically conductive polymers for integration into bioelectronic devices like OECTs. Supplementary Table 4 compiles these materials, enabling direct comparison.

Controlling the swelling degree of the channel material is critical to minimize the deterioration of the film structure that can be detrimental to the performance of OECTs^66^. Therefore, we performed a passive swelling test, which occurs due to water and passive ion diffusion, to comprehend the swelling properties of the biocomposite layer. After immersing a thick-film of biocomposite material (with standard additives) in an aqueous media for 2 days, no visual changes of the specimen were recorded in a fixed geometrical area (Supplementary Fig. 11). On the contrary, the pure Sacran specimen showed anomalous swelling, providing evidence of its high water retention capacity^35^. As previously reported, after the complexation of anionic Sacran with cationic polymer, the swelling degree of the complex was significantly lower than the Sacran-only gel^42^.

After device characterization, the OECTs were subjected to enzymatic biodegradation using the microbial enzyme proteinase K, which is commonly employed to promote enzymatic hydrolysis of PLA^67^. The PLA substrate facilitated the rapid degradation of OECT device at different time intervals (Fig. 6d). The degradation process was evaluated by calculating the weight loss percentage after incubation in PBS and the subsequent removal of the soluble fraction. The accelerated degradation method resulted in approximately ~46% weight loss of the specimen after 13 days (Fig. 6e), with complete degradation expected within a month. In comparison, as reported by Fumeaux et al*.*, PEDOT:PSS OECTs on PLA were exposed to degradation test in soil at 55 °C^24^. However, no visible physical degradation was observed for 3 weeks, and only PEDOT:PSS layer begin to broke down into fragments after a month.

The disposable OECTs presented in this work are fabricated using environmentally benign gold electrodes, a PLA substrate, and PEDOT:Sacran biocompatible channel material. The selected materials used in these devices are suitable for the manufacturing of transient bioelectronics. To assess the biocompatibility of PEDOT:Sacran biocomposites, we conducted cytotoxicity tests following the ISO 10993-5 guidelines. Briefly, the cell medium was applied to wells containing testing samples and a commercial plastic chamber as a control. After 24 h, the medium extract was collected. L929 cells were seeded at 50,000 cells per well in a standard 24-well plate and incubated for 24 h. The medium was then replaced with the collected extracts, and the wells were cultured in medium extracts for an additional 24 h before performing a LIVE/DEAD biocompatibility/cytotoxicity assay using flow cytometry. As shown in Fig. 6f, the reused medium from the studied samples had no adverse effect on cell viability after 24 h. Notably, the viability of cells exposed to PEDOT:Sacran samples was slightly higher than that of the control cells. All samples exhibited high biocompatibility in accordance with according ISO standard testing.

## Discussion

This study presents the successful development of ultrathin, flexible, and biodegradable OECTs utilizing a novel PEDOT:Sacran bio-nanocomposite as the channel material. Sacran’s high molecular weight and anionic functional groups enable its effective integration with PEDOT. The fabricated OECTs demonstrated high transconductance values, maintaining performance even under mechanical deformation. Furthermore, the study highlights the environmental sustainability of transient OECTs by demonstrating their degradability on PLA substrates *via* enzymatic hydrolysis. These findings offer a promising strategy for the design and fabrication of next-generation bioelectronic devices that combine high performance with eco-friendliness. The demonstrated approach paves the way for further exploration of conductive biopolymer-based OECTs in biomedical and environmental applications, addressing the growing demand for sustainable and flexible electronic systems.

The OECTs demonstrated in this work utilize relatively simple device architectures. Future studies may explore optimized configurations through miniaturization and advanced lithography techniques to enhance device performance. Furthermore, improving the intrinsic conductivity and morphology of the Sacran-based composites will require continued material optimization and refinement of processing methods. Finally, to ensure suitability for long-term applications, extended studies under physiologically and environmentally relevant conditions are essential.

## Methods

### Materials

3,4-Ethylenedioxythiophene (EDOT, 99%) was purchased from Thermo Fisher Scientific, while iron (III) *p*-toluenesulfonate hexahydrate (FeTos) was obtained from Sigma-Aldrich. The polysaccharide Sacran was extracted from *Suizenji Nori* cyanobacteria by Maiko Okajima and Tatsuo Kaneko in Japan and transferred to Austria. The solution additives 4-dodecylbenezenesulfonic acid (DBSA), glycerol and (3-glycidyloxypropyl) trimethoxysilan (GOPS) were acquired from Fluka, Carl Roth and Grolman, respectively. Commercial poly(3,4-ethylenedioxythiophene): poly(styrenesulfonate) (PEDOT:PSS, Clevios^TM^ PH 1000) was obtained from Heraeus Electronics GmbH (Hanau, Germany). Prior to usage, the PEDOT:PSS dispersion was filtered through a polyvinylidene fluoride filter with a pore size of 0.45 µm. All other reagents were used as received without further purification. The proteinase K from *Tritirachium album* was purchased from Thermo Fischer Scientific.

Purified 18 MΩ ultrapure water was obtained by the Arium® Mini laboratory water system.

### Extraction of Sacran

Sacran was extracted from *Aphanothece sacrum (A. sacrum)* by the following procedure^31,32,68^. The crude *A. sacrum* biomaterials were freeze-thawed and washed in distilled water at least three times to remove any water-soluble matter. The biomaterials were further washed five times using a large amount of isopropanol with shaking to remove hydrophobic pigments such as chlorophylls. The isopropanol-washed samples were then put into a 0.1 M NaOH and heated to around 100°C and agitated for 4 h to yield the transparent solution. The solution was neutralized with HCl until the pH decreased to 8.0–9.0 and then was filtered. The filtrate was concentrated by a rotary evaporator to create a highly viscous solution. The viscous solution was slowly poured into 60% isopropanol to precipitate a white fibrous material. The extracted yield of Sacran was very high ca. 70 wt % in dried materials of *A. sacrum*.

The absolute molecular weight, *Mw*, was measured by multi-angle static light scattering (MALLS; detection angle from 15° to 40°) and reported previously^43^. From Zimm-Berry plot with an error of 1.4%, the absolute *M*w, radius of gyration, *R*_*g*_, and second virial coefficient, A_2_, for sacran were estimated at 2.35 × 10^7 ^g mol^−1^, 402 nm, and 4.53 × 10^−4^ mole cm^3^ g^−2^, respectively. Sacran was mainly composed of various monosaccharides such as Glc, Gal, Man, Xyl, Rha, and Fuc with a composition of 25.9, 11.0, 10.0, 16.2, 10.2, and 6.9, as a result of GC analyses^32^. Sacran was sulfated in 10 mol% (CHN S elemental analyses) and had a carboxylic acid group in 22 mol% (carbazole-sulfuric acid method (525 nm)) to monosaccharide residues^32^.

### Synthesis of PEDOT:Sacran

The synthesis, applied for the PEDOT:Sacran dispersion, follows the procedure described by Matura et al.^29^. First, a Sacran dispersion in a total concentration of 1 mg mL^−1^ was prepared in 18 MΩ water, whereby the dispersion was vigorously stirred at room temperature for 2 h as well as sonicated in an ultrasonic bath. Next, EDOT monomer was injected in the different respective weight-to-weight (w/w) ratios of monomer to Sacran (1:1, 2.5:1, 5:1, and 7.5:1) followed by 2 h of additional stirring. Subsequently, 2 molar equiv. of iron (III) p-toluene sulfonate hexahydrate (with respect to EDOT) was added into the mixture. The dispersion was heated to 60 °C for 20 h under continuous stirring. After completion of the oxidative chemical polymerization indicated by the dark blue color of the solution, the dispersion was diluted with MΩ water, centrifuged at a speed of 5500 rpm for 3 min and re-dispersed in fresh MΩ water. This process was repeated four times until a neutral pH was reached. Then, the dispersion was sonicated for 30 min followed by a final consecutive centrifugation and re-dispersing step. Next, the dispersion was dialyzed for 24 h by changing the water periodically. Thereby, a dialysis membrane with a molecular weight cut-off (MWCO) of 12–14 kDa was used. The purified biocomposite dispersion was then directly used for the preparation of the OECT devise in an approx. concentration of 10 mg mL^−1^.

To test the redispersability, the dispersed solution of the PEDOT:Sacran was dried by vaporizing the water, yielding a dried solid biocomposite powder. For the preparation of sample dispersion with a concentration of 10 mg mL^−1^ the solid PEDOT-Sacran powder was re-dispersed in a respective amount of MΩ water and sonicated with a UP50H high-power processor (Hielscher Ultrasound Technology) at 60 W with a 0.6 s pulse for 10 min.

*Synthesis of PEDOT:Tosylate* was performed by following the same preparation procedure as for PEDOT:Sacran. The oxidative chemical polymerization was also performed using only EDOT with iron (III) *p-*toluene sulfonate as the oxidant in 2 molar equiv. Here, no Sacran was included. The tosylate (Tos) anion from the oxidant served as a doping counterion and stabilized the oxidized cationic PEDOT.

### Material characterization

*Attenuated total reflectance–Fourier-transform infrared (ATR-FTIR)* spectra were recorded utilizing a Bruker Vertex 80 FTIR spectrometer equipped with a Bruker Platinum diamond attenuated total reflectance (ATR) unit. The materials and dispersion were measured after drying.

*X-ray photoelectron spectroscopy (XPS)* measurements were performed on a Theta Probe XPS-system (Thermo Fisher, GBR), which features a monochromated Al-Kα X-ray source with an energy of 1486.6 eV. The spot size on the sample surface was 400 μm in diameter. The hemispherical analyzer was set to a pass energy of 20 eV for the recorded high-resolution (HR) scans at an energy step size of 0.05 eV. The data acquisition and evaluation, as well as the control of the device, are performed via a software package (Avantage) provided by the system manufacturer. For the XPS characterization, the original stock dispersions (PEDOT:Sacran and PEDOT:PSS) were used without further additives, whereby the dispersions were drop-cast on glass slide.

*Zeta potential and particle size measurements* were conducted using a Malvern Zetasizer Nano ZSP (Malvern Instruments Ltd., Worcestershire, UK). The dynamic light scattering (DLS) method and electrophoretic light scattering (ELS) method were utilized for determining both particle size distribution and zeta potential. The DLS measurements via intensity were performed at 25 °C, placing 1.1 mL of the sample into a disposable cuvette (DTS0012) for the particle size, and 1 mL of sample into folded capillary cells (DTS1070) for the zeta potential determination. To prepare the sample dispersions, 10 µL of the original stock dispersion was diluted with 2 mL of MΩ water and then sonicated for 10 min using an ultrasonic processor set to 60 W with a 0.6-s pulse. Afterwards, the sample dispersions were further diluted transferring 1 mL of the prepared sample into 2 mL of MΩ water. This final sample was sonicated again under the same conditions and then at least 5 measurements were recorded for each type of measurement. The reference PEDOT:PSS dispersions were filtered through a 0.45 µm PTFE syringe filter after the dilution and sonication steps.

The zeta potential results were evaluated through 6 measurements (PEDOT:PSS), 16 (Sacran), 13 (PEDOT:Sacran, 1:1), 12 (PEDOT:Sacran 2.5:1), 24 (PEDOT:Sacran, 5:1), 24 (PEDOT:Sacran, 7.5:1) and 6 (PEDOT:Tos.), respectively. Higher particle size values (above 1000 nm) were dismissed due to possible aggregation effects leading to miscalculations. The zeta potential of 0.59 mV is recorded for the dispersant, MΩ water as control.

*Thermal gravimetric analysis (TGA)* data was recorded using a TGA 4000 Thermogravimetric Analyzer (Perkin Elmer) with a temperature program of heating from 30 °C to 900 °C with 10 °C min^−1^. The 1.5–3.8 mg sample was loaded into the cleaned crucible and then inserted into the instrument. For the TGA measurements, the PEDOT:Sacran with different wt. ratios as well as the reference materials, PEDOT:Tos and Sacran, were tested separately.

### Thin-film characterization

For the characterization of the materials, we used glass substrates (1.5 cm × 1.5 cm) and ITO-coated glass (1.27 cm × 1.27 cm), which were cleaned in an ultrasonic bath with a 2% (v/v) aqueous solution of Hellmanex III, deionized water, acetone and isopropanol at 50 °C for 20 min each. Before further usage, the substrates were blow-dried with N_2_ gas and then treated with oxygen plasma at 100 W for 5 min in a Plasma Etch PE-24 plasma cleaner. For the absorption spectroscopy and conductivity samples of composites, a mixture of 94.5% (v/v) dispersion (PEDOT:Sacran in different weight ratios or PEDOT:PSS), 0.5% (v/v) DBSA, and 5% (v/v) glycerol was prepared, whereby subsequently 1% (v/v) GOPS was added into the dispersion. The composite dispersions were then deposited via drop-casting and via spin-coating methods on glass substrates for conductivity and absorbance measurements, respectively.

The conductivity of the films was measured using an Ossila four-point probe station with supplied software for evaluation.

*Absorption spectroscopy* was performed with a Lambda 1050 UV/Vis/NIR spectrometer (Perkin Elmer) with a 3D WB detector module (Perkin Elmer).

*Contact angle tests* were performed to evaluate the surface hydrophobicity of all sample biocomposites. Multiple 18 MΩ water droplets were measured at room temperature using an Ossila Contact Angle Goniometer and the average values were determined.

*Scanning electron microscopy (SEM)* analyses were conducted using the Thermo Scientific Scios 2 DualBeam instrument with an accelerating voltage of 5 kV. The PEDOT:Sacran dispersions (without additives) were deposited via spin-coating on clean ITO-coated glass substrates for the SEM measurements. The micrographs were taken at ×100,000 magnification. Prior to analysis, the samples were sputtered with gold (Au).

### OECT device fabrication and characterization

The source-drain electrode pattern (channel length: *L* = 60 µm, channel width *W* = 2 mm) was prepared by fixing cleaned glass substrates onto a shadow mask (thickness = 200 µm) to reduce the shadowing effect during the metal deposition process. 10 nm chromium (Cr) and 100 nm gold (Au) were deposited sequentially by thermal evaporation at a pressure of 1–5 × 10^−7^ mbar. Here, Cr was utilized to improve the adhesion of Au to the substrate. Afterwards, the device channel was prepared by depositing a thin-film of the respective sample dispersion (PEDOT:Sacran or PEDOT:PSS) with additives, as mentioned above, via spin-coating using 900 rpm for 40 s, followed by 1200 rpm for 10 s. The area around the channel, as well as the contacts, were carefully wiped by using water-wetted cotton swabs before annealing the substrates at 120 °C for 30 min. In order to increase the film thickness, the spin-coating procedure was repeated whereby additionally a short annealing step of 2 min at 120 °C was employed instead of the final annealing step at 120 °C for 30 min. To deposit a thicker channel layer (approx. 10 μm thick), we drop-cast dispersions and then dried for 30 min at 100 °C followed by a final annealing step of 120 °C for 20 min.

To complete the devices, a laser-cut polymer well of 3M^TM^ VHB^TM^ Tape (acrylic adhesive with a conformable acrylic foam core) was fixed on the substrates to secure the aqueous electrolyte. For the device characterization, 20 µL of phosphate buffered saline (PBS) solution (50 mM, pH 7.4) was used, and a silver/silver chloride (Ag/AgCl) electrode was employed as a non-polarizable gate electrode. All steady-state current-voltage measurements were performed under ambient conditions using an Agilent model E5273A.

A Bruker Dektak XT profilometer with a stylus force of 2 mg was used to measure the film thickness.

Microscope images for the channel geometries were recorded utilizing a Nikon Eclipse LV100 ND microscope in bright-field mode.

### Fabrication of ultrathin and biodegradable devices

To create ultrathin flexible devices, a 1.4 µm thick PET (Mylar® 1.4 CW02) foil was adhered on the glass slide. The glass substrates measuring 25.4 × 27 mm were thoroughly cleaned in an ultrasonic bath for 15 min using a series of solutions: a 2% volume solution of Hellmanex detergent in deionized water, followed by deionized water, acetone, and isopropanol. Next, a polydimethylsiloxane (PDMS Sylgard 184 Silicone Elastomer), solution was prepared by mixing the PDMS base and hardener in a 10:1 weight ratio, diluted with n-hexane at a 1:1 weight ratio. The cleaned glass substrates were coated with a thin layer of PDMS at 4000 rpm for 30 s and then annealed at 125 °C for 15 min. This process promotes adhesion between the PET foil and the glass substrate through van der Waals forces. After securely adhering the PET foil to the glass/PDMS surface, the samples were subjected to an additional annealing step at 115 °C for about 15 min to enhance the adhesion of the thin foil. Finally, the glass/PET substrates were cut into three pieces measuring 25.4 mm × 9 mm using a diamond glass cutter.

To prepare the biodegradable OECTs, a 20 µm thick biaxially oriented polylactic acid (BoPLA Folien NTSS NT, Pütz GmbH + Co. Folien KG) film was applied to 25.4 mm by 9 mm glass substrates using double-sided Kapton tape (3M^TM^). Additionally, the biodegradable molded gelatine wells were used to create fully degradable OECTs.

The OECT devices on PET substrates were completed by applying the same processing steps mentioned above for glass samples. For the OECTs on PLA, the dispersions were drop-cast onto the PLA/glass substrate and then dried and annealed at 100 °C for 20 min, followed by 110 °C for 20. Afterwards, the PET and PLA foils were peeled off and transferred onto a flexible apparatus to measure the device characteristics in initial/compressible and bendable mode. Double-sided acrylic elastomer tapes (3 M VHB) were used as a reliable adhesive supporting platform during device measurements with mechanical strain.

*Compression tests* were conducted using OECT devices prepared on ultrathin PET foil. To evaluate device performance under initial and compression states, a sticky thick acrylic elastomer (3 M VHB) was used. The tape was fixed in an apparatus where it could be stretched to increase its length and then relaxed. The device was placed onto the tape while it was in a stretched state. This setup allowed the device to be compressed when the tape was relaxed by a certain percentage of its length. Multiple tests were conducted, where devices were measured in their initial state, at 5% compression, and at 10% compression relative to the tape length. The results present the statistical distribution of measurements for at least four devices per condition. Characterization was performed under ambient conditions using an Agilent E5273A.

3D map of compressed PET morphology. The wrinkled morphology of the compressed PET foil was analyzed using a Bruker DektakXT stylus profilometer over a scanned area of 2 × 2 mm with 500 lines of surface mapping.

*Tensile tests:* Dumbbell-shaped samples of ultrathin PET and PLA were fabricated for uniaxial tensile testing. The tests were conducted at room temperature using a uniaxial tensiometer (Zwick Roell Z005, 100 N load cell) with a 2 mm/min strain rate.

*Swelling tests* were performed by depositing multiple layers of the biocomposite dispersion with additives as used for the OECT devices via drop-casting. The thick layer of material was then dried for 2 h at 60 °C and then annealed at 120 °C for 30 min. The obtained biocomposite (~20 µm thick) pieces were then put into glass petri dishes and photographed. Afterwards, the pieces were fully immersed in water. Pictures were recorded after 10 min, 1 h, 1 day and 2 days to observe swelling behavior by evaluating geometrical changes. The swelling properties of pure Sacran were investigated by stepwise adding MΩ water (with addition of total water volume of 4 mL, 7 mL, 10 mL, 13 mL, 15 mL, and 20 mL) to the 96 mg Sacran in a crystallization dish. After each additional step, a picture was taken from the swollen Sacran. Finally, after the addition of a total of 20 mL MΩ water, the dish was removed to show the hydrogel structure as well as the absence of liquefaction.

*Biodegradation tests* of PLA-based OECTs were carried out via enzymatic hydrolysis to significantly accelerate the degradation rate. OECT device sample was placed in a vial filled with 5 mL of phosphate buffer saline (0.05 M, pH 8.0) containing 2 mg of proteinase K and 0.1 mg of sodium azide as an anti-mildew and antibacterial agent. The vials are kept in the reservoir well on a warming plate at 37 °C. The buffer/enzyme system was changed every 48 h to restore the original enzymatic activity^67^. The weight of the sample was measured periodically on dry basis during the entire process. The sample was withdrawn from the vial at certain time intervals, washed with distilled water, dried, weighed, and then re-immersed into the degradation media. The percentage weight loss (*WL%*) is calculated by the equation:

2 $${W}_{L} \% =\frac{\left({W}_{i}-{W}_{f}\right)}{{W}_{i}}\cdot 100$$

where *W*_*i*_ and *W*_*f*_ are the respective initial and the final mass of the specimen.

### In vitro biocompatibility analysis

#### Thin-film preparation

For the biocompatibility of the materials, 94.5% (v/v) dispersions of PEDOT:Sacran (5:1), PEDOT:Sacran (7.5:1), and PEDOT:PSS were separately mixed with 0.5% (v/v) DBSA and 5% (v/v) glycerol, whereby subsequently 1% (v/v) GOPS was added for adherence on the glass substrates. The glass slides (1 × 1 cm) were cleaned in an ultrasonic bath with a 2% (v/v) aqueous solution of Hellmanex III, deionized water, acetone and isopropanol at 50 °C for 20 min each. The substrates were dried with N_2_ gas and treated with oxygen plasma at 100 W for 5 min in a Plasma Etch PE-24 plasma cleaner. The respective material dispersions were deposited via spin-coating at 900 rpm for 60 s. The samples were annealed at 140 °C for 1 h for cross-linking GOPS.

#### Cell culture and cell viability (*ISO 10993-5 guidelines*)

Cell line L929 (adipose mouse fibroblasts) was purchased from American Type Culture Collection (ATCC, Manassas, VA, USA) and was cultivated in DMEM (Life Technologies, Carlsbad, CA, USA). The culture medium contained (final concentrations in medium): 10% fetal calf serum (FCS), L-Glutamine (2 mM), PenStrep (5 U penicillin, 50 µg streptomycin/mL). Cells were incubated at 37 °C and under a 5% CO_2_-enriched atmosphere. The LIVE/DEAD assay was performed using a BD FACSVerse flow cytometer (BD Biosciences, San Jose, CA, USA). Heat-killed cells that were incubated at 60 °C for 30 min before measurements were used as a positive control.

After incubation (24 h), we collected the supernatant (500 µL, washed the cells with phosphate-buffered saline (PBS; 0.1 M, pH 7.4; 500 µL), detached the cells with 0.25% trypsin-EDTA solution, and, after 5 min, we resuspended the cells in 750 µL of a culture medium (final volume 2000 µL). There was no volume discarded during the preparation (even PBS for washing was collected), so we obtained information about all cells in our samples. Then, the cells were incubated with PI (10 µg/mL) and calcein-AM (50 µM diluted in DMSO) for 30 min in the dark. Finally, the fluorescence signal was measured on a flow cytometer using the first two scatters (Forward scatter channel vs. Side scatter channel) and then two fluorescence channels (red FL 2: ex. 488/em. 700 nm and green FL 3: ex. 488/em. 527 nm. A red signal due to propidium iodide revealed dead cells that had lost membrane integrity, whereas a green signal indicated live cells with active intracellular esterases that could catalyze the non-fluorescent calcein-AM to highly fluorescent green calcein. Three independent experiments were performed and mean and standard deviation ± (SD) were calculated.

## Supplementary information

Supplemental Information

## Data Availability

The data that support the findings of this study are available within the article, its Supplementary Information, or from the authors.
