# Supplementary material for: Algal polysaccharide Sacran-based conductive nanocomposites for ultrathin flexible and biodegradable organic electrochemical transistors
Source: Npj Flex Electron. 2025 Jun 13;9(1):56. doi: 10.1038/s41528-025-00436-1 (PMC12165848; doi:10.1038/s41528-025-00436-1)
Supplement: Supplementary file 1 — Supplemental Information [file 41528_2025_436_MOESM1_ESM.pdf]

Supplementary Information

Algal polysaccharide Sacran-based conductive nanocomposites for ultrathin flexible and biodegradable organic electrochemical transistors

Katharina Matura[1], Christoph Putz[2], Sarka Hradilova[3], Katerina Polakova[3], Mihai Irimia-Vladu[1], Maiko Okajima[4], Tatsuo Kaneko[4], Martin Kaltenbruner[2], Niyazi Serdar Sariciftci[1], Serpil Tekoglu[1]*

[1]Linz Institute for Solar Cells (LIOS) and Institute of Physical Chemistry, Johannes Kepler University Linz, Altenbergerstrasse 69, A-4040, Linz, Austria

[2]Division of Soft Matter Physics and Institute of Experimental Physics, Johannes Kepler University Linz, Altenbergerstrasse 69, A-4040, Linz, Austria

[3]Czech Advanced Technology and Research Institute (CATRIN), Regional Centre of Advanced Technologies and Materials (RCPTM), Palacký University Olomouc, Šlechtitelů 27, 779 00 Olomouc, Czech Republic

[4]School of Chemical and Material Engineering, Jiangnan University, 1800 Lihu Ave, Wuxi 214122, China

**Author e-mail address: serpil.tekoglu@jku.at*

List of Figures

Supplementary Figure 1. Main molecular structure of *Aphanothece sacrum* polysaccharide, Sacran.

Supplementary Figure 2. Contact angle measurement of 18 MΩ water on pure glass and on a layer of PH1000 (PEDOT:PSS).

Supplementary Figure 3. UV-vis-NIR absorption spectra of thin films of the PEDOT:Sacran composite for different weight ratios.

Supplementary Figure 4. XPS surveys of PEDOT:Sacran biocomposites in different initial weight ratios of EDOT:Sacran. Peak deconvolution of PEDOT:Sacran (weight ratio 5:1) for N1s, Fe2p, and O2s.

Supplementary Figure 5. Comparison of exemplary steady-state electrical characteristics (transfer and output curves) of OECTs with PEDOT:Sacran (different weight ratios) channel material.

Supplementary Figure 6. Steady-state electrical characteristics (transfer and output curves) for OECTs with PEDOT:Sacran (weight ratio 5:1) as channel material on PET foil. Device characterization of reference OECTs with PEDOT:PSS on PET foil and glass substrate.

Supplementary Figure 7. Gate characteristics of OECTs with PEDOT:Sacran (weight ratio 5:1) as channel material on glass substrate, PET, and PLA foils.

Supplementary Figure 8. The stress-strain curve showing the mechanical properties of PET and PLA samples.

Supplementary Figure 9. Ultrathin flexible OECT on PLA substrate is mounted on human skin with various compression ratios.

Supplementary Figure 10. A device photo and transfer characteristic of PEDOT:Sacran-based fully degradable OECTs on PLA foil.

Supplementary Figure 11. Swelling test for PEDOT:Sacran free-standing film and Sacran fibers.

List of Tables

Supplementary Table 1. Comparison of the hydraulic diameter (D_h), zeta potential (ζ -potential) and polydispersity index (PDI) of PEDOT:Sacran composite before and after redispersing.

Supplementary Table 2. The survey scan results including atom percentages of the PEDOT:Sacran biocomposites for different weight ratios.

Supplementary Table 3. Flexible and stretchable OECTs for bioelectronic applications.

Supplementary Table 4. Conductive biodegradable materials for bioelectronic applications.

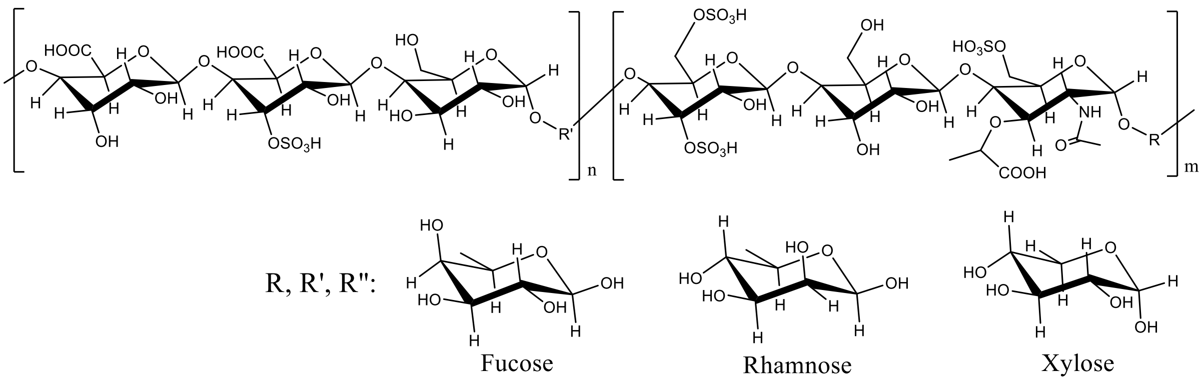

Supplementary Figure 1. Main molecular structure of *Aphanothece sacrum* polysaccharide, Sacran.

Supplementary Table 2. Comparison of the hydraulic diameter (D_h), zeta potential (ζ -potential) and polydispersity index (PDI) of PEDOT:Sacran composite before water removal by rotary evaporator and after redispersing the biocomposite in the same volume of water. The average values are given with the respective standard deviation.

	Material	EDOT: Sacran weight ratio	ζ -potential / mV	D_h / nm	PDI	n
before	PEDOT:Sacran	5:1	-42.0 ± 1.12 (n=24)	635 ± 77	0.348 ± 0.078	12
after	PEDOT:Sacran	5:1	-44.4 ± 1.25 (n=24)	691 ± 45	0.420 ± 0.076	12

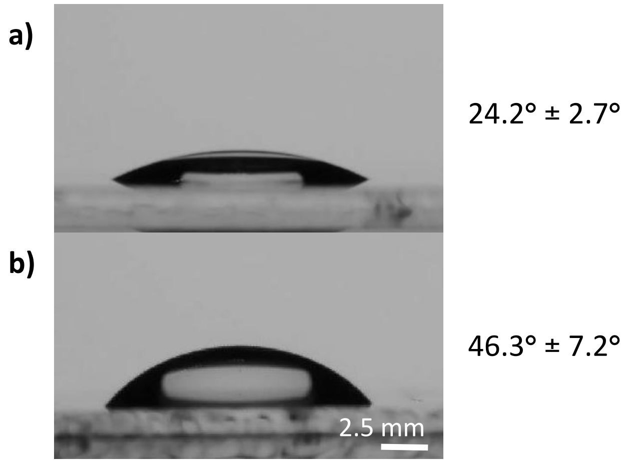

Supplementary Figure 2. Contact angle measurement of 18 MΩ water **a)** on pure glass and **b)** on a layer of PH1000 (PEDOT:PSS) using a dispersion made of 94.5% (v/v) PH1000, 0.5% (v/v) DBSA, 5% (v/v) Glycerol and finally 1% (v/v) GOPS with respect to the volume of the total dispersion. Contact angle measurements were performed on three different substrates. For each measurement, the left and right contact angles were recorded as separate values, resulting in a total of six values included in the statistical evaluation.

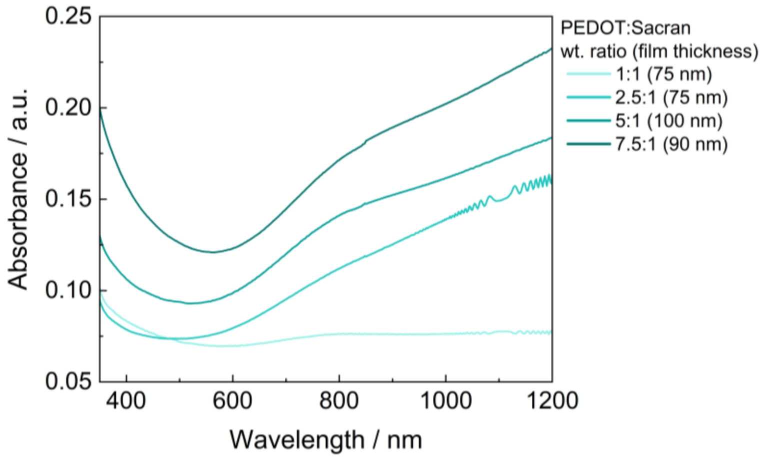

Supplementary Figure 3. UV-vis-NIR absorption spectra of thin films of the PEDOT:Sacran composite for different weight ratios.

Supplementary Table 2. The survey scan results including atom percentages of the PEDOT:Sacran biocomposites for different weight ratios.

PEDOT:Sacran weight ratio	Name	Peak / eV	Height / CPS	FWHM / eV	Area (P) / CPS eV	Atomic percentage /%
1:1	O1s	531.1	14074.28	3.82	58770.08	38.6
	C1s	284.77	6964.43	4.04	30880.47	54.8
	S2p	163.35	768.78	4.84	5566.77	5.7
	Fe2p	710.91	448.1	6.3	7084.63	0.9
2.5:1	O1s	532.59	11202.57	4.08	50108.09	33.9
	C1s	286.15	6610.78	4.16	30339.28	55.5
	S2p	164.38	1470.32	4.37	7519.49	8.0
	Si2p	102.1	211.92	4.56	1214.88	2.6
5:1	O1s	531.08	14844.25	3.63	59636.57	37.4
	C1s	284.59	7025.06	3.95	30466.67	48.3
	S2p	162.61	973.98	4.12	4646.82	12.9
	N1s	101.72	457.46	3.3	1880.91	1.3
7.5:1	O1s	531.05	9343.9	3.95	40989.73	27.6
	C1s	284.11	7295.01	4.22	33978.57	61.7
	S2p	162.47	2150.67	3.98	10163.66	10.7

Residual content of Si2p in the sample (2.5:1) can be traced back to the glass substrate used for the measurement.

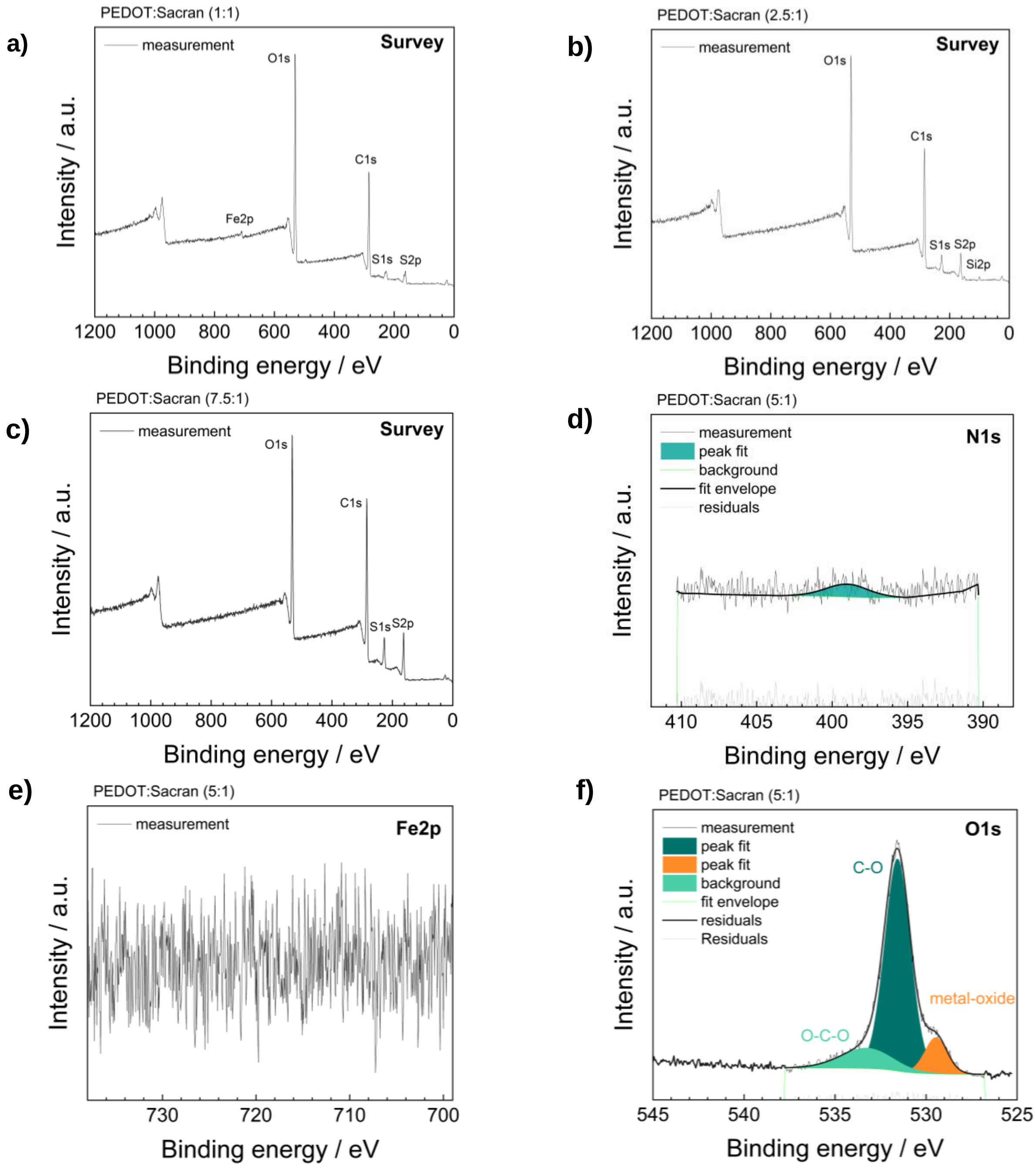

Supplementary Figure 4. XPS results from survey scans of PEDOT:Sacran biocomposites in different initial weight ratios of EDOT:Sacran: **a)** 1:1, **b)** 2.5:1, **c)** 7.5:1. Peak deconvolution of PEDOT:Sacran (weight ratio 5:1) for **d)** N1s and **e)** Fe2p and **f)** O2s.

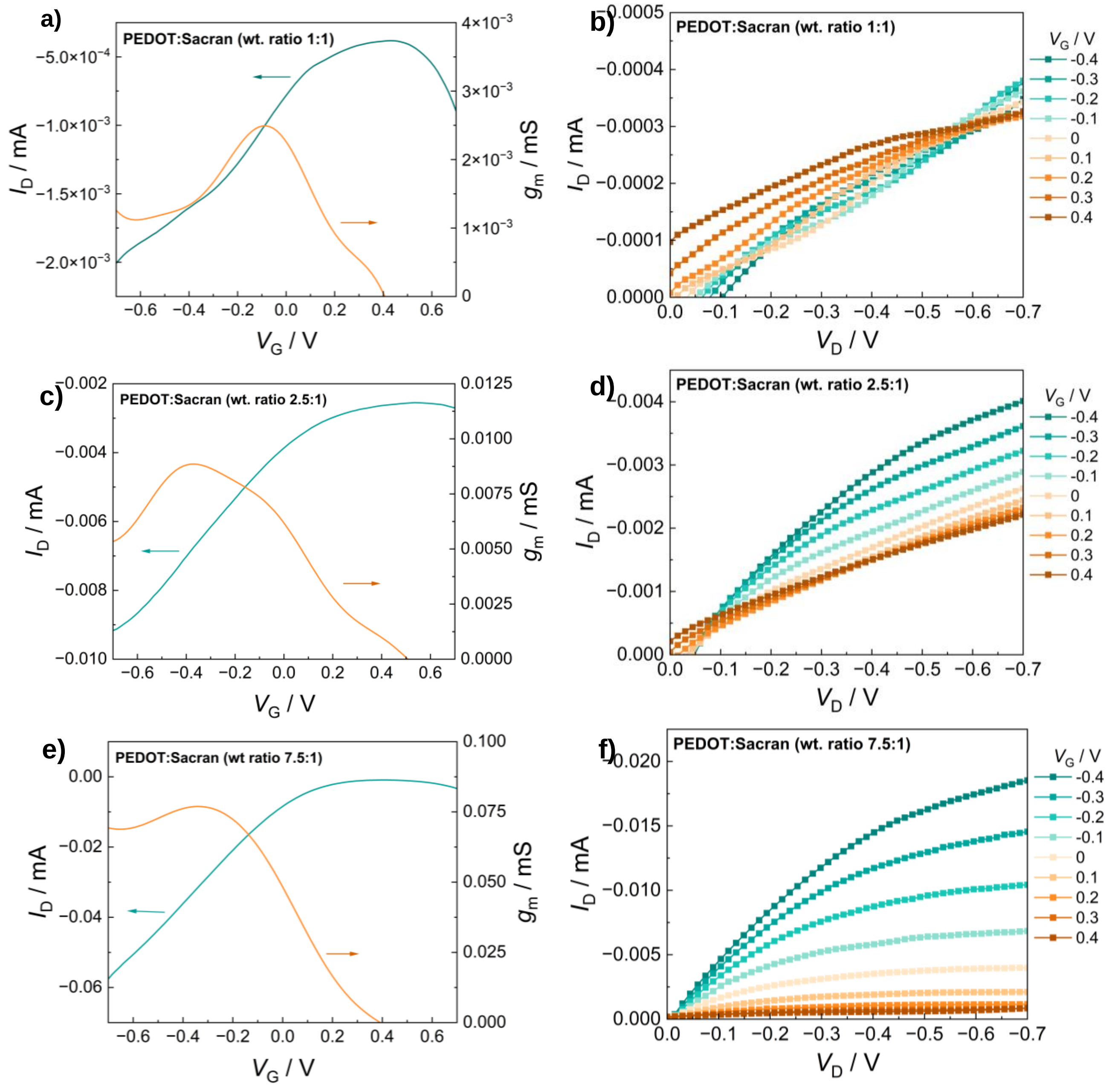

Supplementary Figure 5. Comparison of exemplary steady-state electrical characteristics of PEDOT:Sacran-based OECTs. **a), c)** and **e)** Transfer characteristics and the corresponding transconductance curve (smoothening was applied for all transconductance curves: 10 pts FFT) (for V_D = -0.7 V) and **b)**, **d)** and **f)** the corresponding output characteristics for an OECT with the channel being respectively based on a 1:1, 2.5:1 and 7.5:1 wt. ratio PEDOT:Sacran dispersion (for 1:1 *W* = 2.1 mm, *L* = 63 µm, *d* = ~ 550 nm) (for 2.5:1 *W* = 2.1 mm, *L* = 68 µm, *d* = ~ 430 nm), (for 7.5:1 *W* = 2.2 mm, *L* = 51 µm, *d* = ~ 200 nm).

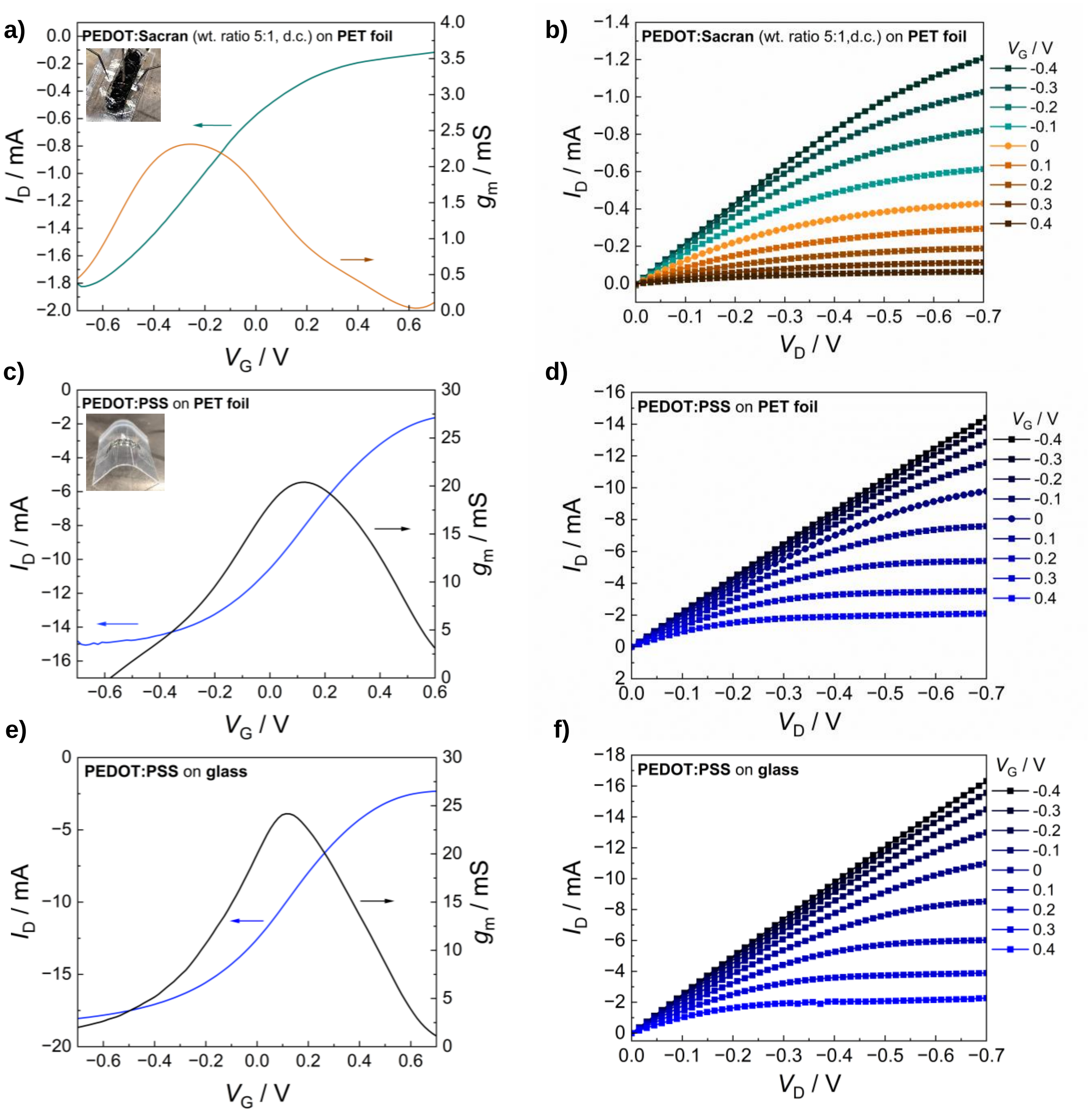

Supplementary Figure 6. Steady-state electrical characterization of PEDOT:Sacran (weight ratio 5:1) based OECTs. **a)** Transfer characteristics and transconductance (V_D = -0.7 V) and **b)** the corresponding output characteristics for an OECT with drop-cast film of PEDOT:Sacran (weight ratio 5:1) as channel layer (*W* = 2.1 mm, *L* = 50 µm, *d* = ~ 5 µm). **c)** Transfer characteristics and the corresponding transconductance curve (for V_D = -0.7 V) and **d)** the corresponding output characteristics for an OECT device based on a PH1000 dispersion, whereby the device geometry was measured before bending the device (*W* = 2.1 mm, *L* = 62 µm, *d* = ~ 300 nm). **e)** Transfer characteristics and the corresponding transconductance curve (for V_D = -0.7 V) and **f)** the respective output characteristics for an OECT device on glass based on a PH1000 dispersion (*W* = 2.1 mm, *L* = 61 µm, *d* = ~ 250 nm).

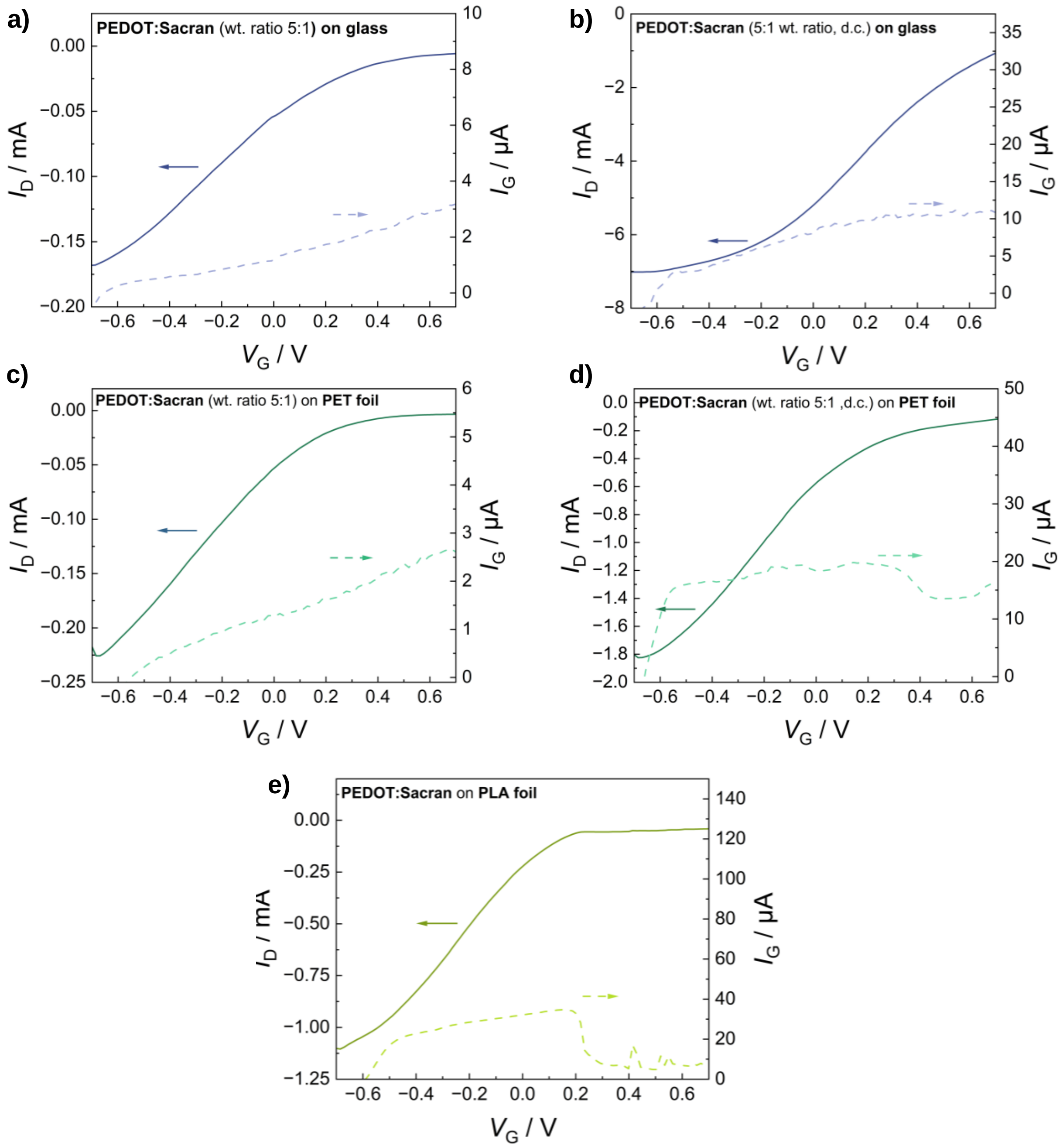

Supplementary Figure 7. Comparison of I_D and I_G curves (V_D = -0.7 V) for PEDOT:Sacran (weight ratio 5:1) based OECTs: **a)** spin-coated device on glass (W = 2.1 mm, L = 65 µm, d = 480 nm), **b)** drop-cast device on glass (W = 2.2 mm, L = 47 µm, d = ~ 7.5 µm), **c)** spin-coated device on PET foil (W = 2.2 mm, L = 55 µm, d = ~ 600 nm), **d)** drop-cast devices on PET foil (W = 2.1 mm, L = 50 µm, d = ~ 5 µm) and **e)** drop-cast device on PLA (W = 2.2 mm, L = 35 µm, d = ~ 9.5 µm).

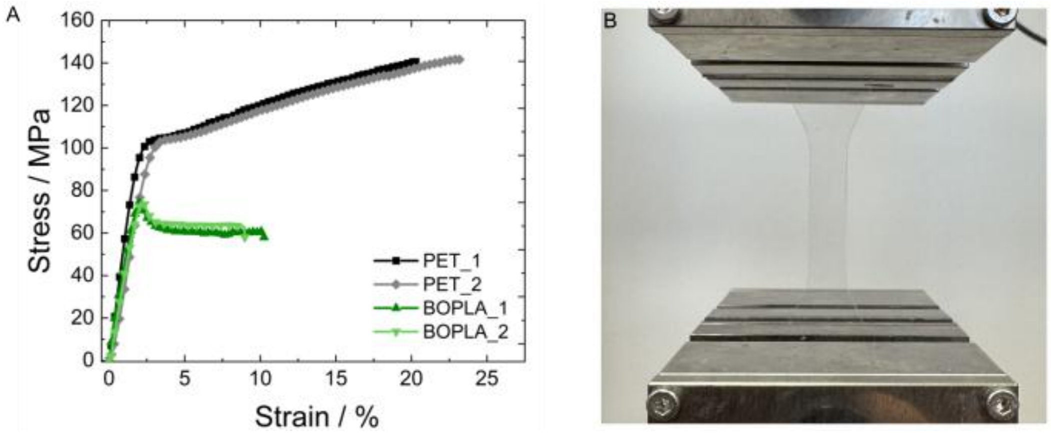

Supplementary Figure 8. a) The stress-strain curve showing the mechanical properties of PET and PLA samples. **b)** A photograph of the specimen during uniaxial tensile tests.

Uniaxial tensile tests for ultrathin PET and PLA samples reveal high tensile strength, reaching stress values above 120 MPa, with a continuous strain-hardening effect beyond yield. In contrast, PLA samples demonstrate a lower yield stress (~70 MPa) and an early plateau in the stress-strain curve, indicating limited plastic deformation. Both thin foils exhibit a yield stress at around 2.5% strain, meaning this can be a limiting factor for stretching without sustaining damage. The PET samples demonstrate approximately twice the strain at failure (20%), whereas PLA exhibits a more brittle behavior with lower elongation.

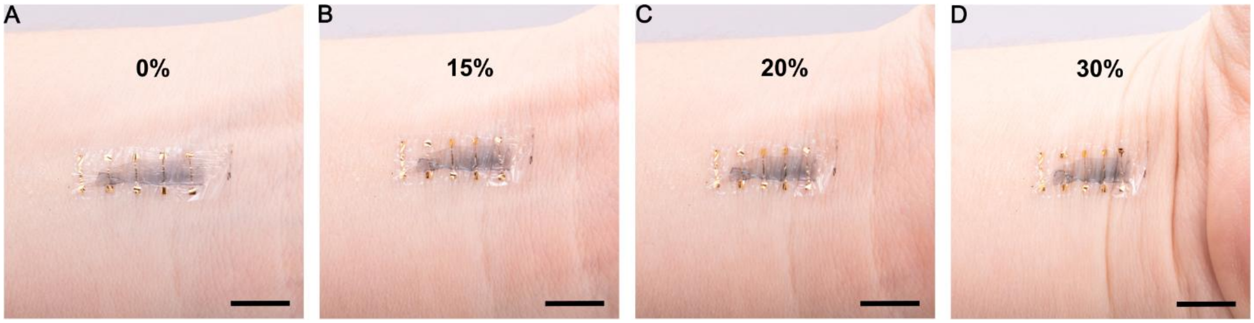

Supplementary Figure 9. Ultrathin flexible OECT on PLA substrate is mounted on human skin with various compression ratios of 0%, 15%, 20%, and 30%.

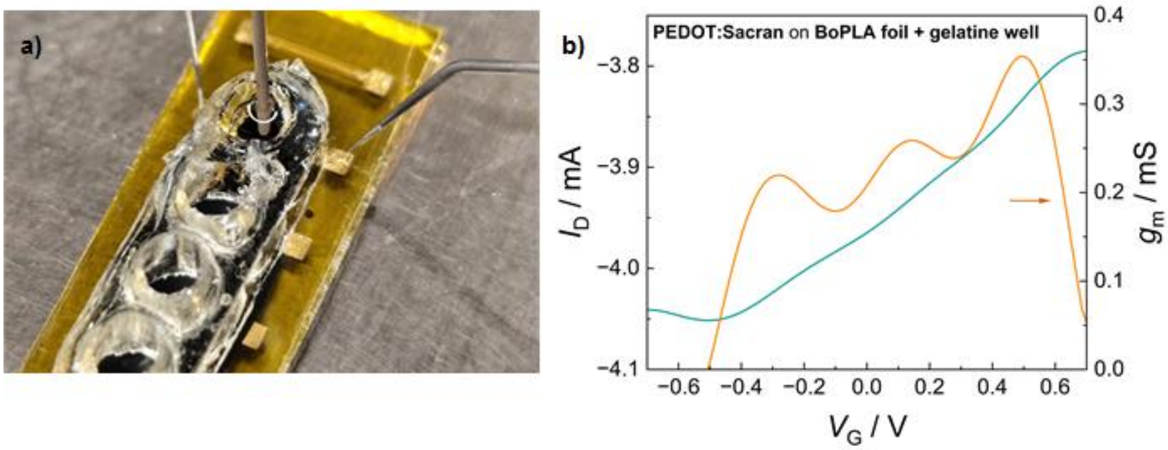

Supplementary Figure 10. a) The photo of PEDOT:Sacran-based (5:1 wt. ratio) OECTs on PLA substrate. The degradable gelatine wells are used to secure PBS electrolyte solution. **b)** The exemplary transfer characteristic of fully degradable OECT and its corresponding transconductance (for *W* = 2.2 mm, *L* = 34 µm, *d* = ~ 9.6 nm), whereby for the transconductance a smoothing was performed (10 pts FFT).

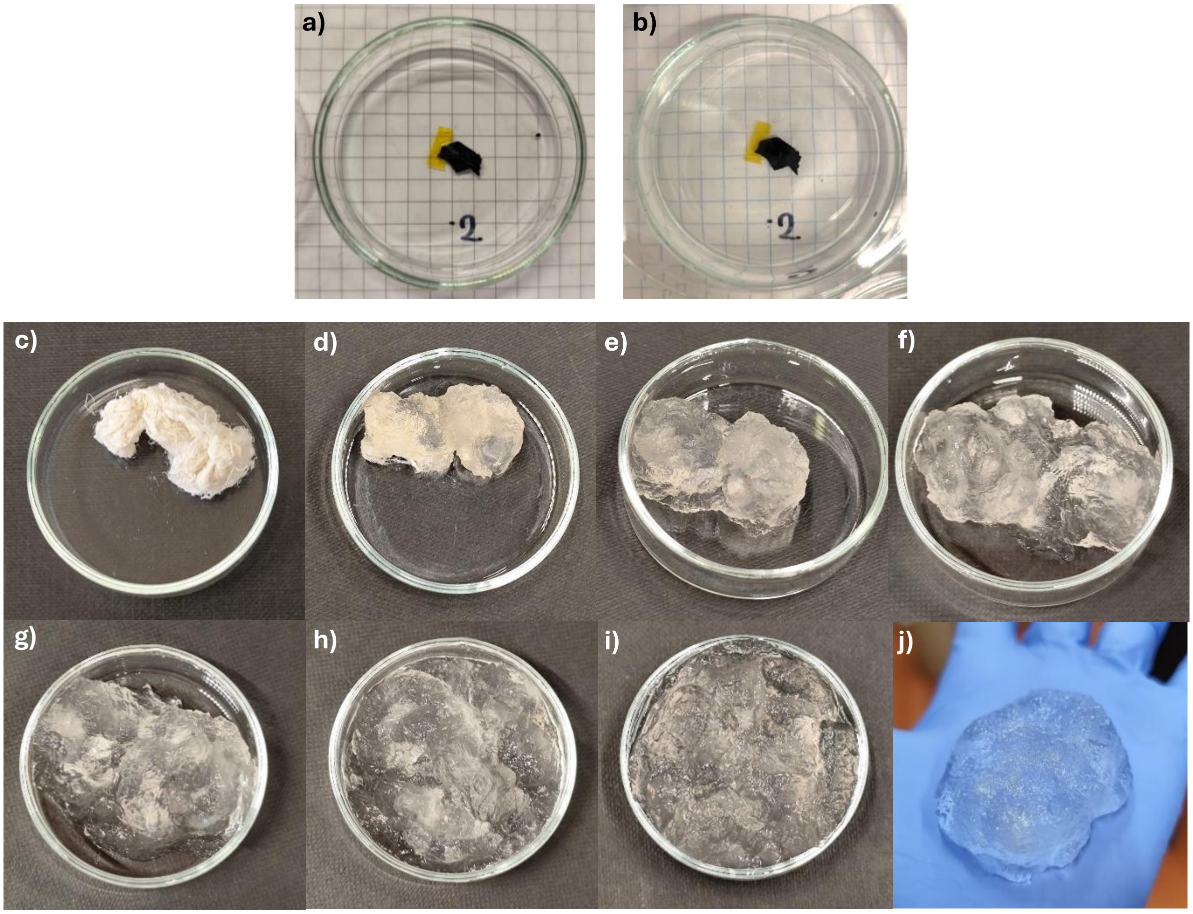

Supplementary Figure 11. Swelling test of a piece of drop-cast PEDOT:Sacran composite with standard additives (0.5% (v/v) DBSA, 5% (v/v) glycerol and 1% (v/v) GOPS with respect to the volume of the total dispersion) **a)** before and **b)** after two days being immersed in 18 MΩ water. Addition of water to Sacran with **c)** initial dry Sacran fibres with addition of total water volume of **d)** 4 mL, **e)** 7 mL, **f)** 10 mL, **g)** 13 mL, **h)** 15 mL and **i)** 20 mL. **j)** Content of **i)** removed from glass dish to show the remaining structural stability and the jelly-like structure of swollen Sacran.

Supplementary Table 3. Flexible and stretchable OECTs for bioelectronic applications.

Channel	Substrate	Electrolyte	Source/Drain	Gate	Flexibility	g_m / mS	*L* / µm	*W* / µm	*d* / µm	g_m(NR) / S cm^{-1}	Ref.
PEDOT:PSS	Parylene	PVA-acrylamide hydrogel	Au	Ag/AgCl	30% stretching strain	1.62	50	50	N/A	N/A	1
PEDOT:PSS	Parylene C (1.2 µm)	No external reservoir due to hydrated ion reservoir	Au	Ag/AgCl	N/A	52.74	5	500	0.3	17.58	2
PEDOT:PSS /[EMIM][TCM]	Polyacrylate (PAR)	0.1 M NaCl	Au	Ag/AgCl	Bending radius: 11 mm	28.7	200	1000	0.8	71.75	3
PEDOT:PSS	Parylene diX-SR (1.2 µm)	PBS	Au	Ag/AgCl	15% extension strain	~ 0.6	15	10	N/A	N/A	4
PEDOT:PSS	PET (177.8 µm)	Cell culture medium	Au	Ag/AgCl	N/A	2.5	6	30	0.1	50	5
PEDOT:PSS	Tattoo Paper on Human Skin	PBS	Au	Ag/AgCl	5% stretching strain	1.5	1000	100000	0.04 – 0.2	3.75 – 0.75	6
PEDOT:PSS	PET (50 µm)	PBS	Pt	PANI/Nafion-graphene/Pt	N/A	0.2	200	6000	0.08	0.83	7
PEDOT:PSS	Parylene	PBS	Au	-	33% compression strain	0.8	200	200	N/A	N/A	8
PEDOT:PSS	PET (150 µm)	Chitosan/Dextran/$LiClO_4$	Ag	Ag	Bending radius: 40 mm	0.416	500	1500	4	0.35	9
PEDOT:PSS	PET (1.4 µm)	0.05 M PBS	Au	Ag/AgCl	Bending radius: 10 mm	20.4	62	2100	0.3	20.1	**This Work**
PEDOT:Sacran	PET (1.4 µm)	0.05 M PBS	Au	Ag/AgCl	Bending radius: 10 mm	2.3	50	2100	~ 5	0.1	**This Work**
PEDOT:PSS	PLGA[a,*]	0.1 M PBS	Au	Pt or Ag wire	Bending radius: 80 µm	3.2	30	1000	0.2	4.8	10
PEDOT:PSS	PLGA[a,*] (12 µm)	0.1 M PBS	Au	Ag/AgCl	Bending radius: ≤ 5 mm	8.67	20	200	0.2	43.35	11
PEDOT:PSS	PLA*	0.1 M PBS	Carbon	Shellac-carbon	N/A	0.247	3000	1000	0.5	14.82	12
PEDOT:Sacran	PLA* (20 µm)	0.05 M PBS	Au	Ag/AgCl	N/A	1.6	35	2200	~ 9.5	0.03	**This Work**

[a] Poly(lactic-*co*-glycolic acid), *Biodegradable substrate.

Supplementary Table 4. Conductive biodegradable materials for bioelectronic applications.

Material	Conductivity / S cm^{-1}	Degradation Conditions	Application	Ref.
PEDOT[a]-HA/PLLA[b]	~ 7 x 10^{-2}	In vitro degradation in PBS	Composite films for in vitro biocompatibility tests	13
PEDOT[a]/carboxymethyl chitosan	~ 5 x 10^{-3}	In vitro enzymatic degradation	Electroconductive hydrogels for nerve tissue engineering	14
PLGA[c]-P3HT[d]	0.1 x 10^{-5}	In vitro degradation in PBS	In vitro biocompatibility of biocomposite nanofibers	15
PPy[e]/chitosan	1 x 10^{-2}	In vitro and in vivo degradation	Porous chitosan scaffold for biomedial applications	16
PPy[e]-PVDF[f]	~ 26	Dissolution in acetone	Nanomembrane for pressure, strain and temperature sensing	17
TPT[g]	6.2 x 10^{-6}	N/A	3D printed flexible scaffolds for biomedical applications	18
PGAP[h]-CSA[i]	~ 2 x 10^{-5}	Degradation in PBS	In vitro biodegradability and biocompatibility	19
P3TMA[j]-PE44[k]	~ 1 x 10^{-4}	Enzymatic degradation	Free-standing nanomembranes for tissue engineering	20
PEA-*g*-TA[l]	~ 8 x 10^{-6}	Enzymatic degradation	Biodegradable polymer for tissue engineering	21

[a] Poly(3,4-ethylenedioxythiophene), [b] Hyaluronic acid/Poly(*L*-lactic acid), [c] Poly(lactide-*co*-glycolide), [d] Poly(3-hexylthiophene), [e] Polypyrrole, [f] Polyvinylidene fluoride, [g] Tetraaniline-b-PCL-b tetraaniline, [h] poly[(glycine ethyl ester) (aniline pentamer) phosphazene], [i] Camphorsulfonic acid, [j] Poly(3-thiophene methyl acetate),[k] Poly(tetramethylene succinate),[l] Poly(ester amide)s-graft-tetraaniline

Supplementary References

1. Lee, H. *et al.* Ultrathin Organic Electrochemical Transistor with Nonvolatile and Thin Gel Electrolyte for Long-Term Electrophysiological Monitoring. *Adv. Funct. Mater.* **29**; 10.1002/adfm.201906982 (2019).

2. Cea, C. *et al.* Enhancement-mode ion-based transistor as a comprehensive interface and real-time processing unit for in vivo electrophysiology. *Nat. Mater.* **19,** 679–686; 10.1038/s41563-020-0638-3 (2020).

3. Wu, X. *et al.* Ionic-Liquid Doping Enables High Transconductance, Fast Response Time, and High Ion Sensitivity in Organic Electrochemical Transistors. *Adv. Mater.* **31,** e1805544; 10.1002/adma.201805544 (2019).

4. Lee, W. *et al.* Nonthrombogenic, stretchable, active multielectrode array for electroanatomical mapping. *Sci. Adv.* **4,** eaau2426; 10.1126/sciadv.aau2426 (2018).

5. Yao, C., Li, Q., Guo, J., Yan, F. & Hsing, I.-M. Rigid and flexible organic electrochemical transistor arrays for monitoring action potentials from electrogenic cells. *Adv. Healthcare Mater.* **4,** 528–533; 10.1002/adhm.201400406 (2015).

6. Zhang, S. *et al.* Hydrogel-Enabled Transfer-Printing of Conducting Polymer Films for Soft Organic Bioelectronics. *Adv. Funct. Mater.* **30**; 10.1002/adfm.201906016 (2020).

7. Liao, C., Mak, C., Zhang, M., Chan, H. L. W. & Yan, F. Flexible organic electrochemical transistors for highly selective enzyme biosensors and used for saliva testing. *Adv. Mater.* **27,** 676–681; 10.1002/adma.201404378 (2015).

8. Park, S. *et al.* Self-powered ultra-flexible electronics via nano-grating-patterned organic photovoltaics. *Nature* **561,** 516–521; 10.1038/s41586-018-0536-x (2018).

9. Sun, B. *et al.* Development of Screen-Printed Biodegradable Flexible Organic Electrochemical Transistors Enabled by Poly(3,4-ethylenedioxythiophene) Polystyrene Sulfonate and a Solid-State Chitosan Polymer Electrolyte. *ACS Appl. Electron. Mater.* **6,** 2336–2348; 10.1021/acsaelm.3c01823 (2024).

10. Campana, A., Cramer, T., Simon, D. T., Berggren, M. & Biscarini, F. Electrocardiographic recording with conformable organic electrochemical transistor fabricated on resorbable bioscaffold. *Adv. Mater.* **26,** 3874–3878; 10.1002/adma.201400263 (2014).

11. Wu, M. *et al.* Ultrathin, Soft, Bioresorbable Organic Electrochemical Transistors for Transient Spatiotemporal Mapping of Brain Activity. *Adv. Sci. (Weinheim, Ger.)* **10,** e2300504; 10.1002/advs.202300504 (2023).

12. Fumeaux, N., Almeida, C. P., Demuru, S. & Briand, D. Organic electrochemical transistors printed from degradable materials as disposable biochemical sensors. *Sci. Rep.* **13,** 11467; 10.1038/s41598-023-38308-1 (2023).

13. Wang, S. *et al.* Fabrication and characterization of conductive poly (3,4-ethylenedioxythiophene) doped with hyaluronic acid/poly (l-lactic acid) composite film for biomedical application. *J. Biosci. Bioeng.* **123,** 116–125; 10.1016/j.jbiosc.2016.07.010 (2017).

14. Xu, C. *et al.* Biodegradable and electroconductive poly(3,4-ethylenedioxythiophene)/carboxymethyl chitosan hydrogels for neural tissue engineering. *Mater. Sci. Eng., C* **84,** 32–43; 10.1016/j.msec.2017.11.032 (2018).

15. Subramanian, A., Krishnan, U. M. & Sethuraman, S. Axially aligned electrically conducting biodegradable nanofibers for neural regeneration. *J. Mater. Sci.:Mater. Med.* **23,** 1797–1809; 10.1007/s10856-012-4654-y (2012).

16. Wan, Y., Yu, A., Wu, H., Wang, Z. & Wen, D. Porous-conductive chitosan scaffolds for tissue engineering II. in vitro and in vivo degradation. *J. Mater. Sci.:Mater. Med.* **16,** 1017–1028; 10.1007/s10856-005-4756-x (2005).

17. Veeralingam, S. & Badhulika, S. Bi2S3/PVDF/Ppy-Based Freestanding, Wearable, Transient Nanomembrane for Ultrasensitive Pressure, Strain, and Temperature Sensing. *ACS Appl. Bio Mater.* **4,** 14–23; 10.1021/acsabm.0c01399 (2021).

18. Prasopthum, A. *et al.* Three dimensional printed degradable and conductive polymer scaffolds promote chondrogenic differentiation of chondroprogenitor cells. *Biomater. Sci.* **8,** 4287–4298; 10.1039/D0BM00621A (2020).

19. Zhang, Q.-S., Yan, Y.-H., Li, S.-P. & Feng, T. Synthesis of a novel biodegradable and electroactive polyphosphazene for biomedical application. *Biomed. Mater. (Bristol, U. K.)* **4,** 35008; 10.1088/1748-6041/4/3/035008 (2009).

20. Armelin, E. *et al.* Biodegradable free-standing nanomembranes of conducting polymer:polyester blends as bioactive platforms for tissue engineering. *J. Mater. Chem.* **22,** 585–594; 10.1039/C1JM14168F (2012).

21. Cui, H. *et al.* Synthesis of biodegradable and electroactive tetraaniline grafted poly(ester amide) copolymers for bone tissue engineering. *Biomacromolecules* **13,** 2881–2889; 10.1021/bm300897j (2012).
